# Natural Bioactive Compounds from Marine Invertebrates That Modulate Key Targets Implicated in the Onset of Type 2 Diabetes Mellitus (T2DM) and Its Complications

**DOI:** 10.3390/pharmaceutics15092321

**Published:** 2023-09-14

**Authors:** Marcello Casertano, Alessio Vito, Anna Aiello, Concetta Imperatore, Marialuisa Menna

**Affiliations:** Department of Pharmacy, University of Naples “Federico II”, Via D. Montesano 49, 80131 Napoli, Italy; marcello.casertano@unina.it (M.C.); alessio.vito@unina.it (A.V.); aiello@unina.it (A.A.); cimperat@unina.it (C.I.)

**Keywords:** marine natural products, diabetes mellitus, metabolic diseases, marine invertebrates, drug discovery, enzymatic targets

## Abstract

Background: Type 2 diabetes mellitus (T2DM) is an ongoing, risky, and costly health problem that therefore always requires new treatment options. Moreover, although several drugs are available, only 36% of patients achieve glycaemic control, and patient adherence is a major obstacle. With monotherapy, T2DM and its comorbidities/complications often cannot be managed, and the concurrent administration of several hypoglycaemic drugs is required, which increases the risk of side effects. In fact, despite the efficacy of the drugs currently on the market, they generally come with serious side effects. Therefore, scientific research must always be active in the discovery of new therapeutic agents. Discussion: The present review highlights some of the recent discoveries regarding marine natural products that can modulate the various targets that have been identified as crucial in the establishment of T2DM disease and its complications, with a focus on the compounds isolated from marine invertebrates. The activities of these metabolites are illustrated and discussed. Objectives. The paper aims to capture the relevant evidence of the great chemical diversity of marine natural products as a key tool that can advance understanding in the T2DM research field, as well as in antidiabetic drug discovery. The variety of chemical scaffolds highlighted by the natural hits provides not only a source of chemical probes for the study of specific targets involved in the onset of T2DM, but is also a helpful tool for the development of drugs that are capable of acting via novel mechanisms. Thus, it lays the foundation for the design of multiple ligands that can overcome the drawbacks of polypharmacology.

## 1. Introduction

Diabetes mellitus (DM) is a metabolic disorder where the body does not produce adequate levels of insulin or does not effectively respond to the insulin being produced. Insulin is secreted by pancreatic β cells when the concentration of glucose rises and is one of the main hormones maintaining the body glucose homeostasis. DM is considered an alarming health problem, which is spreading worldwide with a dramatic incidence, thus burdening not only public health, but also health system expenditures. Over the past three decades, the number of people with diabetes has increased by about 314 million, reaching a total of 422 million cases in 2014, and that number that is predicted to rise. In the same year, it was estimated that 8.5% of adults aged 18 years or older had diabetes. In 2019, diabetes was the primary cause of 1.5 million deaths, and 48% of those deaths occurred in people under the age of 70 years. The incidence of diabetes has increased most rapidly in low- and middle-income countries due to globalization, but also with respect to changes in lifestyle and an ageing population [1,2]. DM is classified into type 1 (T1DM) and type 2 (T2DM); the incidence of T2DM is more common, thereby accounting for about 90% of all the cases of diabetes [2]. Onset T1DM is due to an absolute deficiency of insulin, which occurs when these cells are mistakenly destructed by the immune system. T2DM is mainly caused by insulin resistance and reduced insulin secretion; insulin resistance is when muscle, adipose cells, and liver cells do not respond well to insulin and cannot use glucose from the blood for energy. To make up for this, the pancreas secretes more insulin and, over time, the blood sugar levels rise. This pathological condition, commonly referred as hyperglycemia, thus characterizes T2DM, and it leads to several long-term complications, such as nephropathy, retinopathy, neuropathy, and angiopathy [2,3]. High-calorie dietary habits, ageing, and genetic predisposition are considered to be the main causes that promote the onset of insulin resistance. A relationship between T2DM and obesity has been recognized, and the major basis for this link is the ability of obesity to engender insulin resistance [4]. Insulin resistance, together with dyslipidemia, hypertension, and abdominal obesity, is part of the metabolic syndrome (MS), which includes a cluster of factors that, in total, predisposes one to T2DM [5].

## 2. Common Targets for T2DM Treatment

T2DM can be defined as a multifactorial disease, the pathophysiology of which involves complex signaling and regulatory pathways that have not yet been fully understood. The development of T2DM is primarily caused by alterations in one of the molecular mechanisms involved in tissue insulin synthesis, release, and response; inflammatory processes and hyperglycemia-induced oxidative stress have also been shown to contribute to the disease progress [6]. Strategies for T2DM management certainly include a healthy diet and moderate exercise; moreover, drugs that reduce insulin resistance and obesity are necessary for intervention along the disease state continuum. An important point to emphasize is that monotherapy is often unsuccessful in the management of T2DM; in most cases, combination of several hypoglycemic drugs is required, thus leading to drug–drug interactions and inadequate patient adherence. The main classes of drugs to treat T2DM aim to counteract hyperglycemia through different mechanisms of action. Insulin secretagogues act by stimulating pancreatic β-cells to secrete more insulin; the main representative of this class are sulfonylureas [7]. Another class of currently used drugs are biguanides, which, unlike insulin secretagogues, do not influence the insulin secretion directly, but instead improve the body’s response to natural insulin. These molecules reduce hepatic glucose production and increase glucose uptake and utilization by peripheral tissues [8]. Insulin mimetic sensitizers are agents that help in lowering blood glucose levels; this effect is reached by activating the glucose transporters on muscle and fat cells or by increasing the sensitivity of the body tissues towards insulin. Among the insulin sensitizers, we can include the Peroxisome proliferator activated receptor (PPAR) agonists, which regulate protein and carbohydrate metabolism and maintain the glucose homeostasis. The activation of PPARα affects the glucose metabolism, since it causes a decrease in hepatic gluconeogenesis, whereas this increases the utilization of peripheral glucose. Moreover, PPARγ agonists increase the sensitivity of the cells to insulin, improve glucose uptake by skeletal muscles, and decrease the glucose production by retarding gluconeogenesis. Thus, dual PPARα/γ agonists have been also discovered with synergistic action in maintaining insulin sensitivity and inflammation control, with reduced side effects with respect to using PPARγ agonists alone [9,10].

Over the last few years, new therapy strategies have emerged in the treatment of T2DM, and several other targets involved in the establishment of hyperglycemia and its related complications have been identified; of course, these represent interesting and useful therapeutic targets for research into new ligands to be developed as drugs as well [11,12]. For instance, the approach of reducing the digestibility of carbohydrates by controlling the activity of the hydrolyzing enzymes α-amylase and α-glucosidase is now considered to be a valid prophylactic treatment for T2DM [13]. In fact, α-amylase and α-glucosidase inhibitors can slow the final stages of carbohydrate digestion, thus preventing glucose from entering circulation, and they could be used to control postprandial hyperglycemia. Following these, incretin-mimetic drugs have emerged as important tools in the management of T2DM [14]. These agents exert their effect via the incretin system by specifically targeting the receptor for the incretin hormone glucagon-like peptide 1 (GLP-1), which is one of the metabolic hormones secreted after nutrient intake that increases insulin release (incretins). GLP-1 agonists stimulate the synthesis and secretion of insulin from the β cells of the pancreas and, thus, lead to a decrease in blood glucose levels [15,16]. Dipeptidyl peptidase IV (DPP-IV) inhibitors also can be considered to be incretin mimetics agents. DPP-IV inhibitors are responsible for the degradation of GLP-1; the inactivation of DPP-IV causes an increase in the half-life of GLP-1 and its activity; thus, DPP-IV inhibitors prolong the action of incretins, which reduces glucose production and increases insulin production [17]. Antagonists/inhibitors of the sodium–glucose cotransporter type 2 (SGLT2), which mediate glucose reabsorption from the glomerular filtrate, are another recently discovered class of hypoglycemic drugs. SGLT2 inhibitors, which prevent the reabsorption of glucose, enhance the excretion of glucose in urine, and the glucose level in the blood is maintained [18]. The use of fructose-1,6-bisphosphatase (FBPase) inhibitors [19] is now recognized as a new valid approach for the management of T2DM. FBPase is a rate-limiting enzyme involved in gluconeogenesis that leads to the production of endogenous glucose; reducing this glucose excess in the blood and tissues would relieve the T2DM symptoms. The largely used antidiabetic drug, metformin, has recently been shown to act through FBPase inhibition. The glycogen synthase kinase 3 (GSK-3) is a negative regulator of glycogen synthase; it is a glycosyltransferase that catalyzes glycogen chain elongation and helps in the conversion of glucose to glycogen. GSK-3 selective inhibition in insulin-resistant skeletal muscle causes improvements in insulin-stimulated glucose transport activity that are likely caused by enhanced postinsulin receptor insulin signaling and GLUT-4 glucose transporter translocation [20]. Protein tyrosine phosphatase 1B (PTP1B), which is broadly expressed in various cells and tissues, is one of the main negative regulators of the insulin receptor (IR) signaling pathway. It decreases the phosphorylation of the IR signaling pathway, thereby resulting in insulin resistance in various tissues. PTP1B inhibitors improve the sensitivity of the insulin receptor and have the ability to cure insulin-resistance-related diseases [21]. The reduction of glucose by aldose reductase (AR) in the polyol pathway has been linked to the development of secondary diabetic complications. AR is a cytoplasmic aldo−keto reductase that has been widely investigated as an enzyme that is critically involved in the onset and progression of pathologies associated with DM, where it plays a pivotal role in mediating hyperglycemia-induced oxidative stress and consequent tissue and vascular damage through the NADPH-dependent reduction of glucose to sorbitol and the consequent modifications of both the cellular osmolarity and redox status. Accordingly, AR inhibitors have been shown to prevent or slow the progression of T2DM-associated pathologies [22,23].

Based on the above, it is clear that, although several effective therapeutics are available today, scientific research must always be prompt and active not only in the identification of new active substances for drug discovery and development, but also in the ever-deepening understanding of the key cellular and molecular processes involved in the pathobiology of T2DM to reveal additional molecular targets and, thus, new mechanisms of action (MoAs) for new drugs. Moreover, in the process of finding new therapeutics, the multifactorial nature of T2DM should be considered; it should be kept in mind that the traditional “one-target-one-drug” model is being replaced with a “multitarget” approach that could be particularly beneficial for the treatment of T2DM and could overcome the drawbacks of polypharmacology. In this strategy, molecules with a multitarget profile, that is, molecules that are capable of modulating two or more selected biological targets, are searched for and/or rationally designed. In addition, since a high risk for cardiovascular diseases is usually associated with T2DM, targets involved in both diabetes and cardiovascular disease are particularly attractive as well [24].

### 2.1. Marine Invertebrates as Potential Sources of New Antidiabetic Leads

Now that advances in the field of genomics have made it possible to identify tens of thousands of potential drug targets, the main problem in drug discovery in its early stages is the availability of new chemical scaffolds, and the probability of being successful lies in the number of small molecules that can be used as their modulators. Capturing natural products’ (NPs’) biological activity represents an advantageous alternative to combinatorial chemistry as a resource of chemical diversity that offers particularly favorable features such as huge scaffold diversity and structure complexity. The bioprospecting of natural resources, such as terrestrial plants and/or marine organisms, as well as a sophisticated use of the biologically relevant chemical space offered by (NPs), may provide the key to drive innovation at nearly all stages of a drug discovery process. Natural matrices and NPs have been the first source of the medical remedies used to treat human diseases for thousands of years. NPs have a unique and vast chemical diversity and have evolved to effectively bind to biological macromolecules; moreover, they may serve as biologically validated starting points for the design of focused libraries that might provide protein ligands with enhanced quality and probability [25]. In recent years, interest in the less-explored marine world has significantly grown thanks to technological advances that have made it possible to investigate the biodiversity of this environment. Since the early research efforts focusing on bioactive marine NPs (MNPs), they have arisen as a new and sustainable resource for drug leads, with unprecedented structural motifs and a plethora of interesting biomedical potentialities with novel mechanisms of action [26,27,28,29,30]. In particular, marine invertebrates, such as sponges, tunicates, soft corals, bryozoans, and nudibranchs, are excellent sources of bioactive NPs, which have been proven to be valuable sources for drug discovery and development. In fact, most of the approved commercial marine-based drugs are of a marine invertebrate origin [26,31]. Although, in many cases, the roles of these MNPs in the marine species themselves are still unclear, the ecology of MNPs has shown that many of them are chemical weapons and are potent inhibitors of the physiological processes in prey, predators, or marine organism rivals. Several MNPs have been demonstrated to play a well-defined role as trail markers, sexual attractants, antifouling substances, or antifeedants [32].

Regarding the use of MNPs for the treatment of human diseases, several MNPs isolated from marine invertebrates have been reported to exhibit glycemic control with different mechanisms of action, wherein some of them are suitable for the multitarget approach as well. In this review, we analyzed the literature from the last thirty years on MNPs that were isolated from marine invertebrates, which can bind/modulate one or more of the identified targets for antidiabetic screening. The collected molecules were first categorized according to their mechanism of action and, within the most populated of those categorized, they were further grouped according to their chemical structures. The aim of this manuscript is to gather relevant evidence that the great chemical diversity associated with MNPs can become a key tool that can advance understanding in the field of T2DM research and antidiabetic drug discovery. Indeed, the natural hits described can become useful leads for the development of drugs that can act via novel mechanisms and interact with different targets, thus laying the foundation for the design of multiple ligands that can overcome the drawbacks of polypharmacology. Regardless, the medicinal chemistry approaches to design and develop multitarget agents require an accurate characterization of the pharmacodynamics and pharmacokinetic profile of the ligand, thus taking into account that translating the in vitro IC_50_ and EC_50_ data to in vivo models is more complicated than single-target ligands [33]. This also implies important toxicity issues that, on the other hand, must be considered also in the case of polypharmacotherapy.

Even though natural products’ levels of bioavailability, efficacy, and specificity on binding to targets are still debatable, these potential hindering challenges could be overcome. The marine-derived lead molecules could be used either directly or as structural templates for the development of more effective multitarget antidiabetic drugs. Moreover, they can be used as chemical probes for studying the functioning of specific targets that are involved in the onset of T2DM and its chronic complications.

### 2.2. Search Methodology

A SciFinder and PubMed search was conducted until July 2023 to identify all the studies related to marine natural products that were able to bind/modulate one or more targets involved in the onset of T2DM. The search included review articles, original articles, and communications. The search terms included “diabetes mellitus”, “marine natural products”, and “invertebrates”, which were published in the English language.

## 3. Results

### 3.1. PTP1B Inhibitors

Protein tyrosine phosphatases (PTPs) constitute an enormous and structurally variable superfamily of enzymes which, together with protein tyrosine kinases (PTKs), regulate the phosphorylation rate of tyrosine residues in proteins. Disturbance to the normal balance between PTK and PTP activity, which, therefore, result in an alteration of the level of tyrosine phosphorylation, has been linked to the etiology of metabolic diseases, inflammatory processes, and neoplastic growth [34]. Considering the great success of kinases as targets for drug discovery in recent years, PTPs have been suggested as the next frontier. Among these, protein tyrosine phosphatase 1B (PTP1B) is of particular interest, since it is significantly involved in the development of insulin resistance, which is a characteristic pathological condition in T2DM. PTP1B acts as a negative regulator of insulin action by dephosphorylating the specific residues of phosphotyrosine of both the activated insulin receptor and its substrates, which, thus, interrupt the signaling pathways mediated by the hormone [21]. PTP1B also downregulates the leptin pathway, which is an adipocyte-derived hormone that controls food intake and increases energy expenditure [35]. PTP1B overexpression is strictly related to insulin resistance, and it has been demonstrated that the inhibition or genetic ablation of this phosphatase can improve glucose homeostasis, cellular sensitivity to both insulin and leptin, and resistance to diet-induced obesity, all without inducing hypoglycemia or toxic effects. This evidence paved the way to the development of PTP1B inhibitors as potential safe therapeutic interventions for the treatment of T2DM and obesity [21,36].

**Figure 1:**
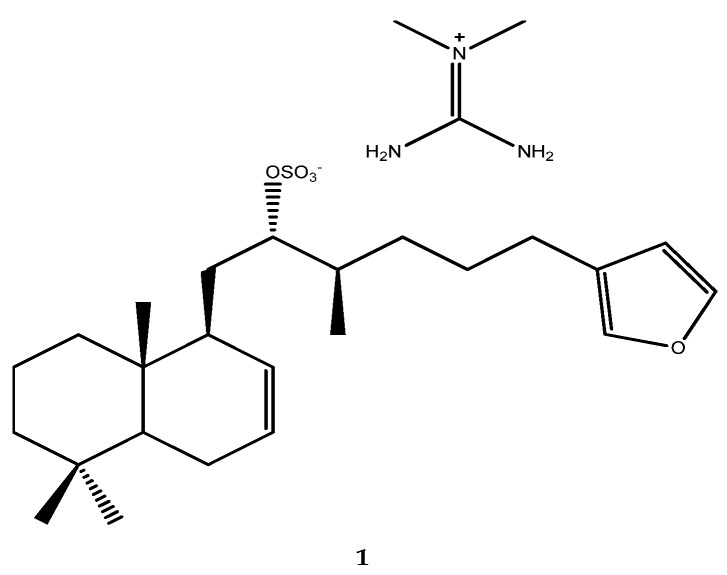
Structure of sulfircin (**1**), the first reported marine-derived PTP1B inhibitor.

More than 300 compounds with inhibitory activity against the PTP1B enzyme that have been isolated from different natural sources are known. The isolation and characterization of sulfircin (compound **1**, Figure 1), an MNP that has been isolated from the sponge *Ircinia* sp. as a PTP1B inhibitor (IC_50_ = 29.8 μmol/L), paved the way to the world of marine invertebrates as a source of PTP1B inhibitors with unusual and peculiar frameworks [37].

#### 3.1.1. Terpenes

Terpenes, which are derived biosynthetically from units of isoprene (C_5_H_8_), are classified sequentially by size as hemiterpenes, monoterpenes, sesquiterpenes, diterpenes, sesterterpenes, triterpenes, tetraterpenes, and polyterpenes.

**Figure 2:**
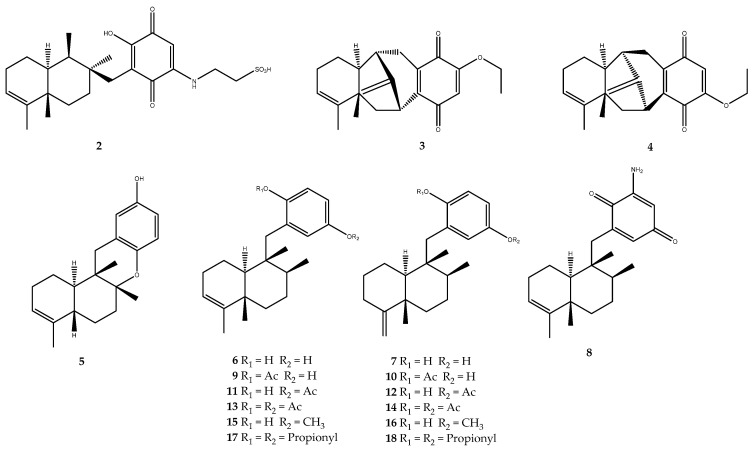
Structures of natural and semisynthetic sesquiterpene quinones/hydroquinones **2**–**18**.

Sponges belonging to the genus *Dysidea* are a valuable source of the compounds that are characterized by sesquiterpenic quinone/hydroquinone moieties, which are often endowed with several biological activities. Dysidine (compound **2**, Figure 2) was isolated from the Hainan sponge *Dysidea villosa* and exerted inhibition activity against PTP1B, with an IC_50_ of 1.5 ± 0.4 μM [38]. A mechanism of action has also been partially outlined for dysidine, which does not involve the involvement of the quinone moiety. In fact, Zhang Y. and coworkers demonstrated that compound **2** inhibited PTP1B without irreversible oxidation or the covalent addition of a quinone moiety. The generation of ROS is not involved in the mechanism of action either. In addition, dysidine displayed a reversible and competitive inhibition against PTP1B, thus additionally acting as a “slow-binding” inhibitor [38].

Dysidavarones A and D (compounds **3** and **4**, Figure 2), which are two new sesquiterpene quinones endowed with the unprecedented “dysidavarane” carbon skeleton, were isolated from the South China Sea sponge *Dysidea avara* and were tested against PTP1B, wherein they showed IC_50_ values of 9.98 μM and 21.6 μM, respectively [38,39].

Avapyran (compound **5**, IC_50_ = 11.0 μM), avarol (compound **6**, IC_50_ = 12.0 μM), neoavarol (compound **7**, 35% inhibition at 32 μM), 3′-aminoavarone (compound **8**, IC_50_ = 18.0 μM), 17-*O*-acetylavarol (compound **9**, IC_50_ = 9.5 μM), 17-*O*-acetylneoavarol (compound **10**, IC_50_ = 6.5 μM), 20-*O*-acetylavarol (compound **11**, IC_50_ = 10.0 μM), and 20-*O*-acetylneoavarol (**12**, IC_50_ = 8.6 μM) were isolated from *Dysidea* sp. collected in Okinawa (Figure 2). All of these natural sesquiterpene quinone/hydroquinone were funneled into a wide screening for the evaluation of the PTP1B activity, and the pharmacological results highlighted compound 10 as the most potent inhibitor of the series [40].

The other IC_50_ values suggested that the avarol-type bicyclic sesquiterpene scaffold appeared to be more favorable for inhibitory activity than the neoavarol-type one. Indeed, the neoavarol monoacetyl derivatives, which are compound **9** (IC_50_ = 9.5 μM) and compound **11** (IC_50_ = 10.0 μM), were more potent than similar derivatives of avarol, mainly compound **10** (IC_50_ = 6.5 μM) and compound **12** (IC_50_ = 6.5 μM). In this work, modifications via the semisynthesis of the simple congeners, avarol (**6**) and neoavarol (**7**), have been performed, on the hydroxy groups on the aromatic system in position 17 and 20, in order to delineate the preliminary SARs (Figure 2). The compounds 17,20-*O*-Diacetylavarol (compound **13**, IC_50_ = 8.8 μM), 17,20-*O*-Diacetylavarol (compound **14**, IC_50_ = 14.0 μM), 20-*O*-methylavarol (compound **15**, 0% Inhibition at 25 μM), and 20-*O*-methylavarol (compound **16**, 50% Inhibition at 31 μM) were inactive, even when compound **14** showed slightly stronger activity than that of neoavarol (**7**). Furthermore, the inhibitory activities of 17,20-*O*-dipropionyl derivatives (compound **17**, IC_50_ = 8.8 μM, and compound **18**, IC_50_ = 9.4 μM) were more active than those of the 17,20-*O*-diacetyl derivatives, thus suggesting the importance of the long chains of the acylic group in the inhibition of the enzyme [40]. This promising evidence outlined drimane-type sesquiterpene hydroquinones as a new chemotype for the development of new PTP1B inhibitors as antidiabetic agents.

**Figure 3:**
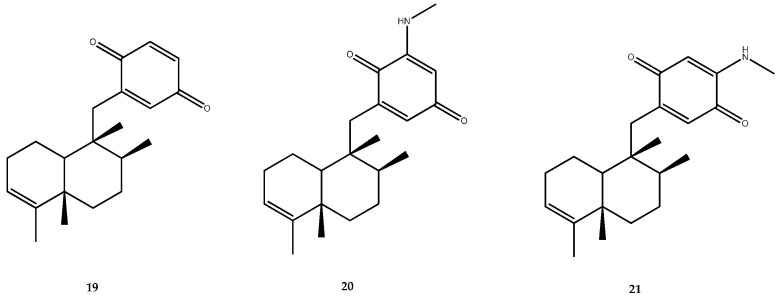
Structures of avarone (**19**) and its methylamino derivatives **20** and **21**.

The chemical investigation of the Aegean sponge *Dysidea avara* by our research group led to the isolation of the already mentioned avarol (**6**), its oxidized form avarone (**19**), 3-(methylamino)avarone (**20**), and 4-(methylamino)avarone (**21**, Figure 3) [41,42]. Those compounds were funneled into a screening for the identification of a dual-type inhibitor of the PTP1B and AR enzyme. As for the inhibitory effect against PTP1B, avarone (**19**) was the most active compound of the series, with an IC_50_ value of 6.7 ± 0.6 μM, while 3-(methylamino)avarone (**20**), 4-(methylamino)avarone (**21**), and avarol (**6**) showed IC_50_ values of 15.2 ± 2.1, 21.6 ± 1.0, and 42.2 ± 18 μM, respectively [41]. The different IC_50_ value obtained for avarol (**6**) compared to the already reported one [40] was due to the difference in experimental conditions for the pharmacological assay performed. These preliminary results outlined that the substitution in position 3 and 4 with a methylamino decreased the activity but not by as much as the reduction of the quinone moiety to hydroquinone, which led to a dramatic decrease in the potency. The further pharmacological characterization of avarone (**19**), which was also identified as an AR inhibitor, highlighted this molecule as a reversible and competitive inhibitor of PTP1B that exerts both insulin-mimetic and insulin-sensitizing activity [41].

**Figure 4:**
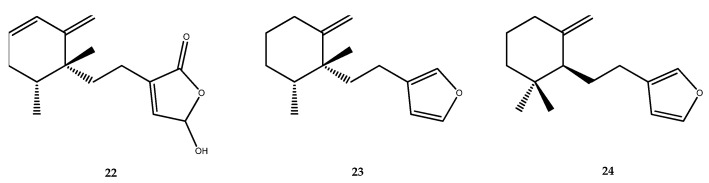
Structure of hydoxybutenolide (**22**), microcionin-4 (**23**), and dihydropallescensin (**24**).

Three sesquiterpenes—hydroxybutenolide (**22**), microcionin (**23**), and dihydropallescensin-2 (**24**, Figure 4)—were isolated and structurally characterized from the Hainan sponge *Dysidea septosa*. These compounds possess a simplified rearranged drimane nucleus and displayed PTP1B inhibitory activities, with IC_50_ values of 8.8, 11.6, and 6.8 µg/mL, respectively [43].

**Figure 5:**
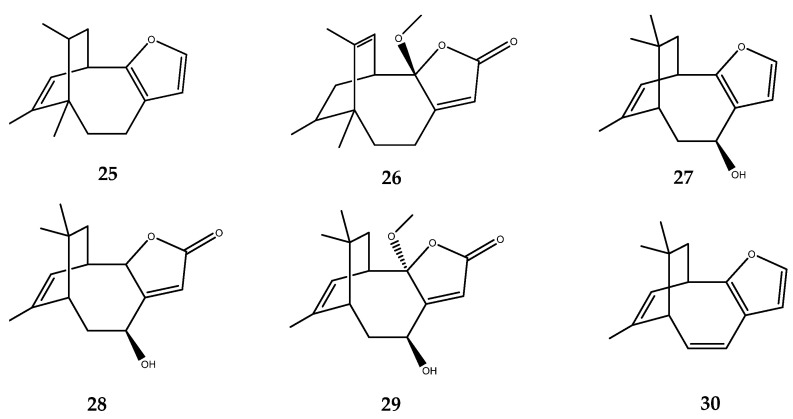
Structures of nakafuran-8 (**25**), O-methylnakafuran-8 lactone (**26**), euryspongins A–C (compounds **27**–**29**), and the semisynthetic compound dehydrospongin A (**30**).

**Figure 6:**
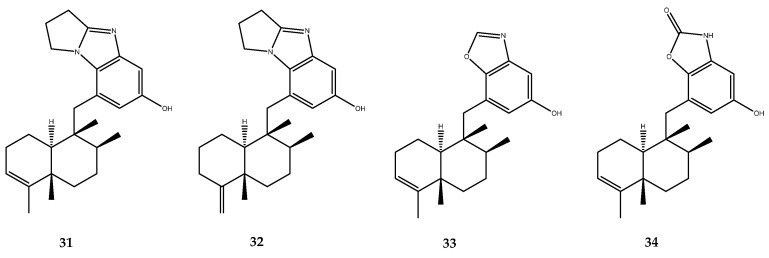
Structures of cinerols A–C (**31**–**33**) and cinerol F (**34**).

Nakafuran-8 (**25**, Figure 5), a 6/8-bicyclic furanosesquiterpene, and O-methylnakafuran-8 lactone (**26**, Figure 6), a 6/8-bicyclic sesquiterpene lactone, were isolated from the sponges *Dysidea septosa* and *Dysidea* sp., respectively. Both compounds showed a strong PTP1B inhibitory effect, with an IC_50_ value of 1.9 µg/mL [43] for compound **25** and 1.58 μM for compound **26** [44].

Moreover, the euryspongins A–C (**27**–**29**, Figure 5) possess the same bicyclic structure, with six- and eight-membered rings of the previous compounds and the furan or β-unsaturated-γ-lactone ring motif. These compounds were isolated from the marine sponge *Euryspongia* sp., which was collected at Iriomote Island, Okinawa. Contrary to the previous compounds, the euryspongins A–C were inactive on PTP1B, while the semisynthetic product, dehydroeuryspongin A (**30**), which was obtained from the dehydration of the parent compound **27**, exerted a potent inhibitory activity, with an IC_50_ of 3.6 μM. Taken together, these results outlined that compounds **27**–**29**, which possess a similar framework to compounds **25**–**26**, were inactive for the presence of the hydroxy group, as was confirmed from the enhanced pharmacological activity of the semisynthetic compound **30**, which lacks this functionality [45].

Wei-Hua Jiao and coworkers isolated from the marine sponge *Dysidea cinerea*, collected in the South China Sea, some sesquiterpenes possessing the driman-type scaffold but which was characterized by a heterocyclic side chain (Figure 6), which showed an interesting inhibitory activity regarding the PTP1B enzyme. Cinerols A (**31**) and B (**32**) presented a rare 5H-pyrrolo [1,2a]benzimidazole moiety and showed an IC_50_ of 3.86 ± 0.45 and 6.63 ± 0.29 μM, respectively. Cinerol C (**33**) was characterized by a rare benzoxazole moiety and displayed an IC_50_ value of 8.82 ± 2.46 μM. Lastly, cinerol F (**34**) was endowed with the uncommon benzoxazolone moiety and exerted the less potent inhibitory effect, with an IC_50_ value of 18.3 ± 0.69 μM [46].

**Figure 7:**
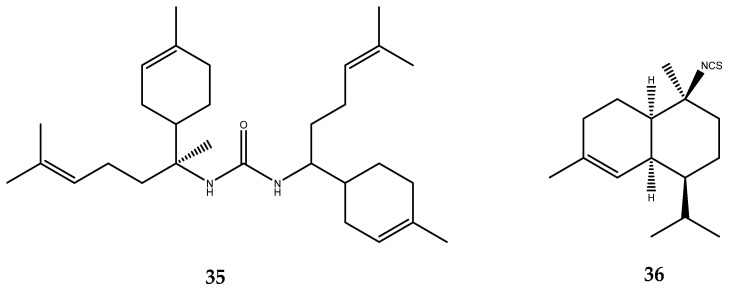
Structures of compound **35** and **36**.

Compounds **35** and **36** (Figure 7) were isolated from the marine sponge *Axinyssa* sp., which was collected at Iriomote Island. Compound **35** possesses a dimeric urea of the bisabolene sesquiterpene moiety and was the most potent inhibitor of the PTP1B enzyme, with an IC_50_ = 1.9 μM, while compound **36** is equipped with an unusual isothiocyanate group, typical of marine natural products, but was less potent, with an IC_50_ of 17 μM [47].

**Figure 8:**
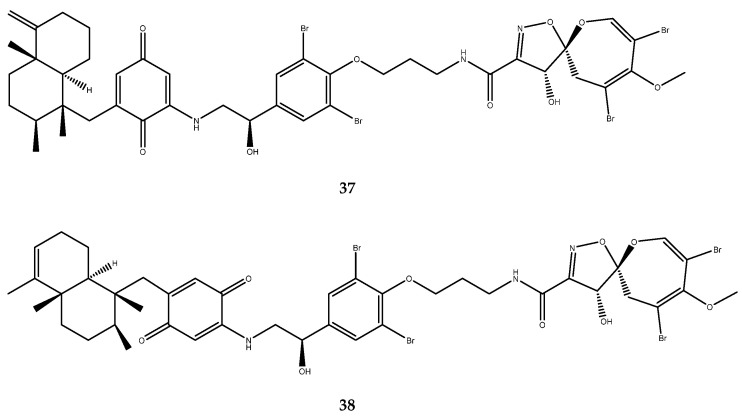
Structures of frondoplysins A (**37**) and B (**38**).

Frondoplysins A (**37**) and B (**38**, Figure 8), which were isolated from the marine sponge *Dysidea frondosa*, are two uncommon sesquiterpenes which display a bioconjugate of a meroterpene and a complex psammaplysin alkaloid structure. Frondoplysins A (**37**) and B (**38**) showed a remarkable potent inhibitory activity, with IC_50_ values of 0.39 ± 0.04 and 0.65 ± 0.03 μM, respectively [48].

**Figure 9:**
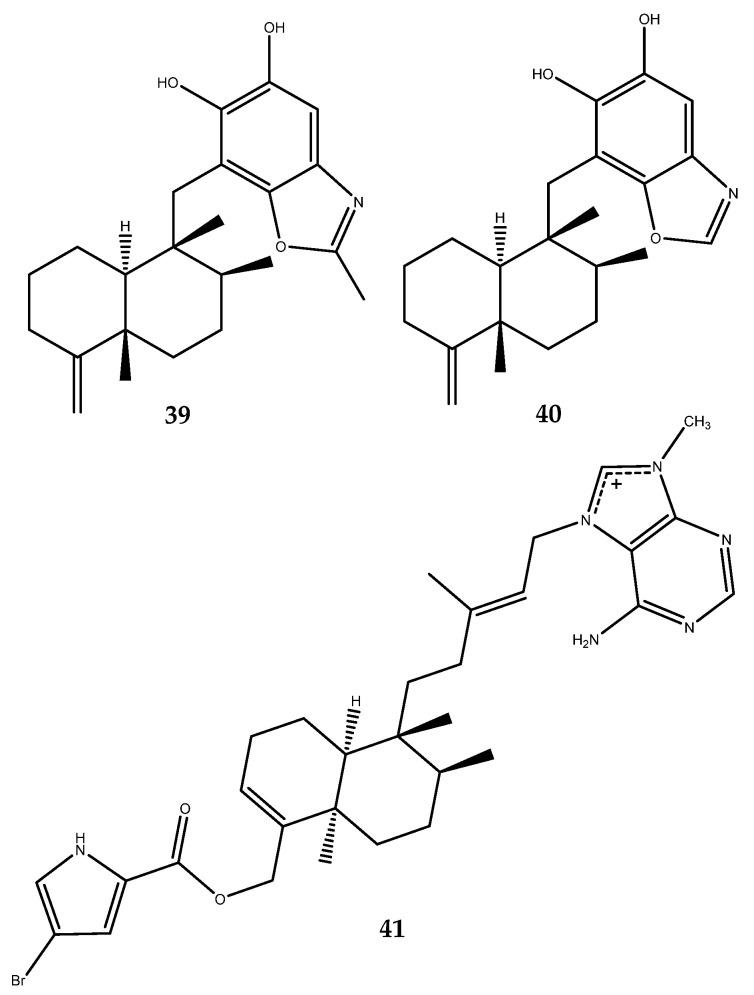
Structures of nakijinol G (**39**) and its inactive congeners nakijinol B (**40**) and agelasine G (**41**).

New meroterpenoids characterized by a drimane-type scaffold linked with a benzoxazole ring were also isolated from *Hyrtios* sp., which is a marine sponge collected from the South China Sea. One of them, nakijinol G (**39**, Figure 9), displayed a good inhibitory activity, with an IC_50_ value of 4.8 μM. An inactive congener of compound **39**, isolated from the same sponge, was reported, nakijinol B (**40**, Figure 9), to outline how the presence of a methyl group on the benzoxazole ring in nakijinol derivatives could increase their PTP1B inhibitory activity [48,49].

Agelasine G (**41**, Figure 9) is an N-methyladenine-derived sesquiterpene, isolated from the Okinawan marine sponge *Agelas nakamurai*, which was determined as a PTP1B inhibitor, with an IC_50_ value of 15 μM. Interestingly, the congener, which lacks bromine on the pyrazole system, was inactive, thereby outlining the importance of this atom in enzyme inhibition [50].

**Figure 10:**
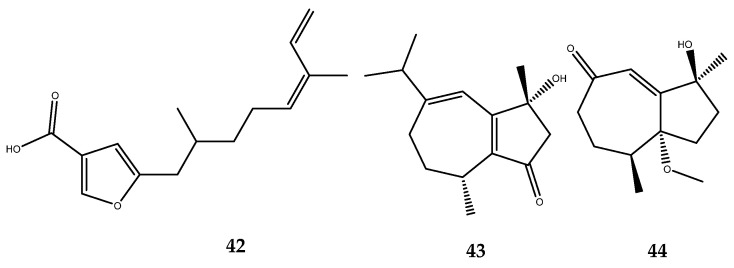
Structures of sesquiterpenes **42**–**44**.

The analysis of the secondary metabolites from the soft coral *Sinularia* cf. *molesta*, which was collected from the Paracel Islands of the South China Sea, yielded a known furanosesquiterpene (**42**) and two new guaiane-type sesquiterpenes (**43** and **44**, Figure 10), which are characterized by a 7/5-bicyclic ring system. These sesquiterpenes were also assessed for their inhibitory activities against PTP1B. The pharmacological results revealed that compounds 42–44 displayed strong inhibitory activities against PTP1B, with IC_50_ values of 1.24, 218, and 344 µM, respectively, which were lower than the positive control (the IC_50_ value of sodium orthovanadate was 881 µM) [51].

**Figure 11:**
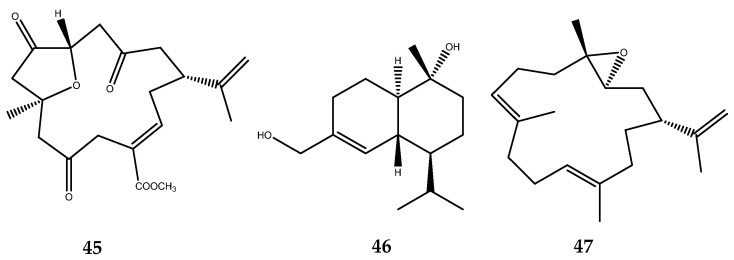
Structures of compounds **45**–**47**.

A new cembranoid, named sinulin D (**45**, Figure 11) [52], as well as two known terpenoids, 15-hydroxy-α-cadinol and (1*R*,3*S*,4*S*,7*E*,11*E*)-3,4-epoxycembra-7,11,15-triene (**46** and **47**, Figure 11) [53,54], were isolated from the Xisha soft coral *Sinularia* sp. Compounds **45**–**47** showed mild target activities against PTP1B, with IC_50_ values of 47.5, 22.1, and 12.5 mM (sodium orthovanadate as the positive control had an IC_50_ = 881 µM) [52].

**Figure 12:**
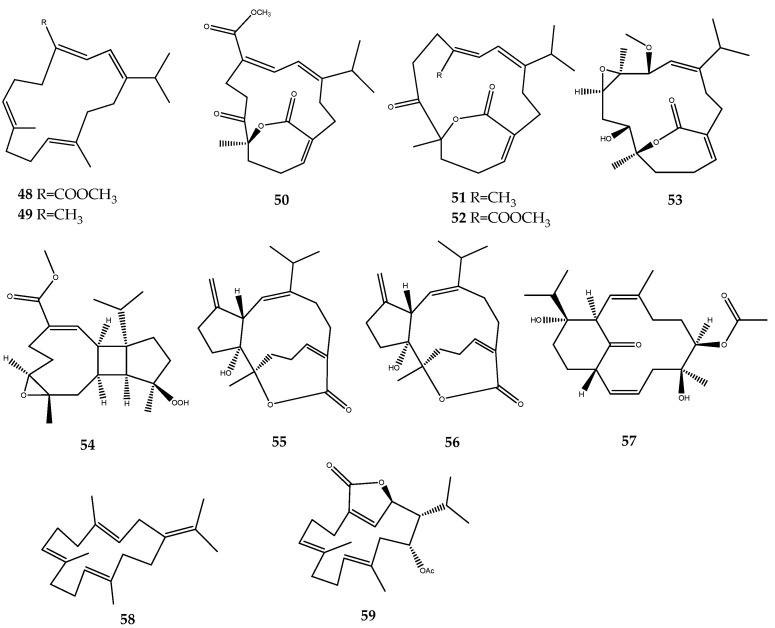
Structures of diterpenoids **48**–**59**.

Many researchers were involved in the bioprospecting of soft corals in the search for new inhibitors of the PTP1B enzyme. The analysis of the secondary metabolites of several samples of *Sarcophyton trocheliophorum* yielded the new PTP1B inhibitors **48**–**59**, which are endowed with a diterpenoid moiety (Figure 12): sacrophytonolide N (compound **48**, IC_50_ = 5.95 µM), cembrene-C (compound **49**, IC_50_ = 26.6 µM), ketoemblide (compound **50**, IC_50_ = 27.2 µM), 4*Z*,12*Z*,14*E*-sarcophytolide (compound **51**, IC_50_ = 15.4 µM), sarcrassin E (compound **52**, IC_50_ = 6.33), sartrolide H (compound **53**, IC_50_ = 19.9 ± 3.13 µM), methyl sarcotroate B (compound **54**, IC_50_ = 6.97 µM), sarsolilide A (compound **55**, IC_50_ = 6.8 ± 0.9), sarsolide B (compound **56**, IC_50_ = 27.1 ± 2.6), secodihydrosarsolenone (compound **57**, IC_50_ = 13.7 µM), (*E*,*E*,*E*)-1-isopropenyl-4,8,12-trimethylcyclotetradeca-3,7,11-triene (compound **58**, IC_50_ = 22.19 µM), and sarcophytonolide I (compound **59**, IC_50_ = 11.26 µM) [55,56,57,58,59,60]. Based on these preliminary data, some SARs could be outlined. Compounds **48**–**54** present a cembrane diterpenoid moiety. The two most active compounds, **48** and **52** of this cluster, share the presence of a dienoate motif, which was believed be responsible of the enhanced activity, as was confirmed from the weak activity of compound **49**, which is completely similar to compound **48** but lacking the ester group [55]. Compound **54** possesses a conjugated ester function with a single double bond, which is unlike the previously mentioned compounds; however, it still maintains a potent inhibitory activity. Furthermore, this is the first PTP1B inhibitor, to our current knowledge, to be endowed with a hydroperoxide function, which is believed to be involved in its mechanism of action [56]. Compound **55** and **56**, instead, are characterized by a capnosane diterpene skeleton. The exomethylene group of the capnosane skeleton in compound **55** could be involved in the inhibition of the enzyme, considering the marked decrease in the activity of compound **56**. In addition, the spatial orientation of the hydroxyl group of compound **56** is essential to guarantee the activity, as was demonstrated by the loss activity of its isolated diasteroisomer at C-10 [59].

**Figure 13:**
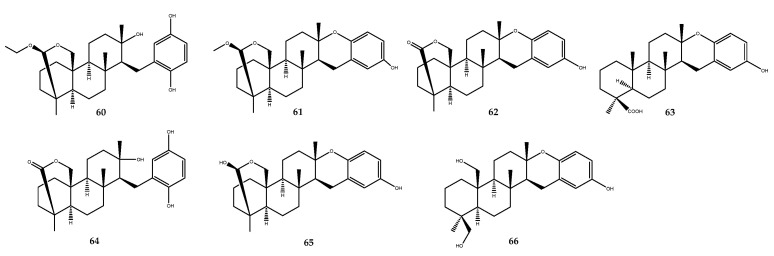
Structures of strongylophorines (**60**–**66**).

The chemical investigation of the Okinawan marine sponge *Strongylophora strongilata* extract led to the identification of diterpenoids called strongylophorines (compounds **60**–**66**, Figure 13) as PTP1B inhibitors: 26-*O*-ethylstrongylophorine-14 (compound **60**, IC_50_ = 8.7 µM), 26-*O*-methylstrongylophorine-16 (compound **61**, IC_50_ = 8.5 µM), strongylophorine-2 (compound **62**, IC_50_ > 24.4 µM), strongylophorine-3 (compound **63**, IC_50_ = 9.0 µM), strongylophorine-8 (compound **64**, IC_50_ = 21.2 µM), strongylophorine-15 (compound **65**, IC_50_ = 11.9 µM), and strongylophorine-17 (compound **66**, IC_50_ = 14.8 µM). Considering the IC_50_ values, some SARs could be outlined. In particular, the compounds endowed with the acetal moiety (compounds **60**, **61**, and **65**) showed stronger inhibitory activities than the lactone derivatives (**62** and **64**) and diol derivative (compound **66**). Structural variations at the aromatic moiety, in contrast, did not influence inhibitory activity against PTP1B [61].

**Figure 14:**
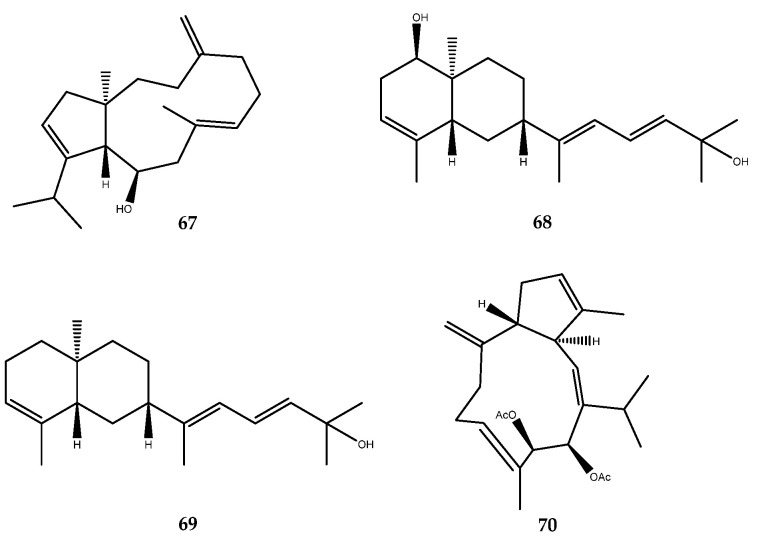
Structures of the diterpene-type compounds **67**–**70**.

Dolabellanes diterpenes, which are characterized by a 5/11-fused bicyclic scaffold, were isolated from the Xisha soft coral *Clavularia viridis*. Among these, clavurol E (**67**, Figure 14) was identified as a PTP1B inhibitor, with an IC_50_ value of 14.5 μg/mL, thus representing the first dolabellane-type diterpenoid inhibitor reported in the literature [62].

Two prenyleudesmane-type diterpenes, **68** and **69**, and a capnosane-type diterpenoid, sinulacetate (**70**, Figure 14), were isolated from the Xisha soft coral *Sinularia polydactyla*. Compounds **68**, **69**, and **70** exhibited a weak inhibitory activity, with IC_50_ values of 75.5 µM, 63.9 µM, and 51.8 µM, respectively [63].

**Figure 15:**
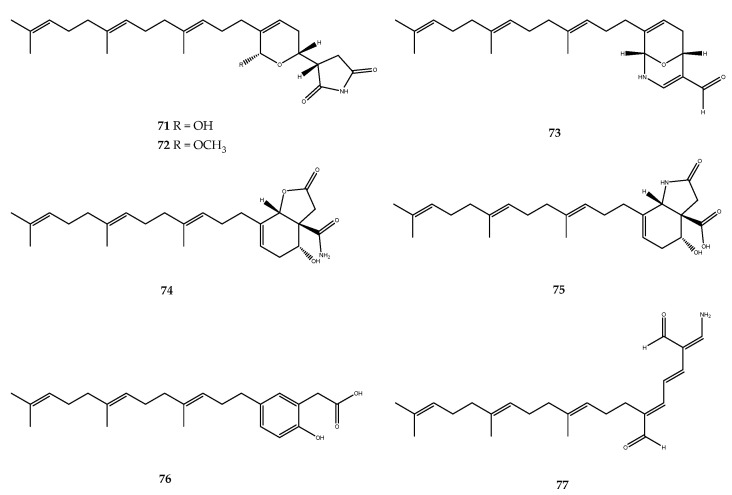
Structure of hippolide derivatives **71**–**77**.

Seven sesterterpenoids, compounds **71**–**77** (Figure 15), were isolated from the South China Sea sponge *Hippospongia lachne*. The most active compounds of the series were compounds **73** and **77**, which showed IC_50_ values of 5.2 and 8.7 μM, respectively. Compounds **74** and **75** displayed weak PTP1B inhibitory activities, with IC_50_ values of 33.0 and 14.0 μM, respectively. In the end, hippolides A (**71**) and B (**72**) exhibited weak PTP1B inhibitory activity, with IC_50_ values of 23.8 and 39.7 μM, respectively [64,65].

**Figure 16:**
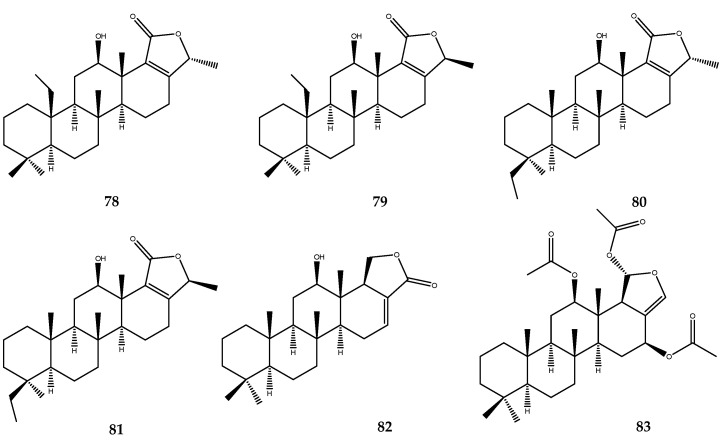
Structures of scalarane-type sesterterpenes: hyattellactones A and B (**78** and **79**), phyllofolactones F and G (**80** and **81**), and compounds **82**–**83**.

Hyattellactones A (**78**) and B (**79**), along with phyllofolactones F (**80**) and G (**81**, Figure 16), were isolated from the Indonesian marine sponge *Hyattella* sp. These compounds are pentacyclic scalarane sesterterpenes with an α,β-unsaturated-γ-lactone ring. Compounds **78** and **80** inhibited PTP1B activity, with IC_50_ values of 7.45 and 7.47 μM, respectively. On the other hand, compounds **79** and **81**, which are (24*S*)-isomers of **78** and **80**, were inactive, thus outlining the essential role of the C-24 methyl spatial orientation [66].

Two scalarane-type sesterterpenes, **82** and **83** (Figure 16), which were isolated from the South China Sea sponge *Hyrtios erecta*, exerted inhibitory activity against the PTP1B enzyme, with IC_50_ values of 19.68 μM and 8.81 μM, respectively [67].

**Figure 17:**
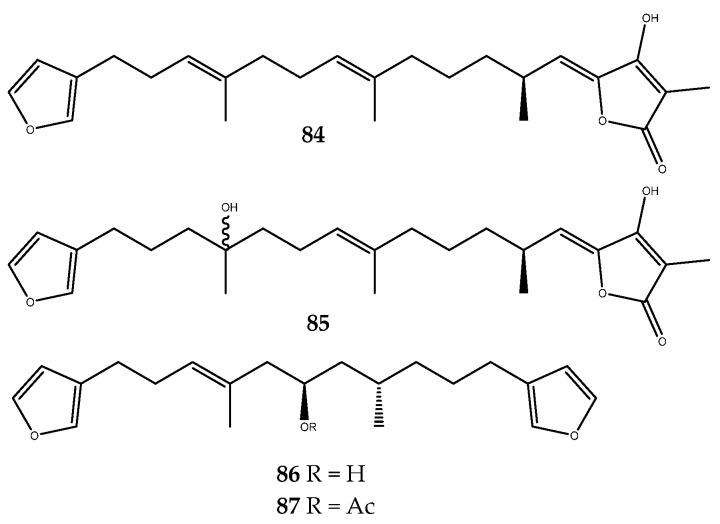
Structures of (7*E*, 12*E*, 20*Z*, 18*S*)-variabilin and (12*E*, 20*Z*, 18*S*)-8-hydroxyvariabilin (**84** and **85**, respectively), as well as furospongin-1 (**86**) and 11-*O*-acetylfurospongin-1 (**87**).

Two furanosesterterpenes, (7*E*, 12*E*, 20*Z*, 18*S*)-variabilin (**84**) and (12*E*, 20*Z*, 18*S*)-8-hydroxyvariabilin (**85**, Figure 17), which were isolated from the Indonesian marine sponge *Ircinia* sp., as well as furospongin-1 (**86**) and its semisynthetic derivative **87** (Figure 17), which were isolated from the Indonesian marine sponge *Spongia* sp., were identified as new types of PTP1B inhibitors, wherein they displayed IC_50_ values of 1.5, 7.1, 9.9, and 9.2 μM, respectively. The stronger activity of the compound **84** than **85** underlines how the hydroxyl group at C-8 leads to a decrease in pharmacological activity. Moreover, the acetylation of C-11 hydroxy group of **87** does not affect the inhibition of PTP1B [68].

**Figure 18:**
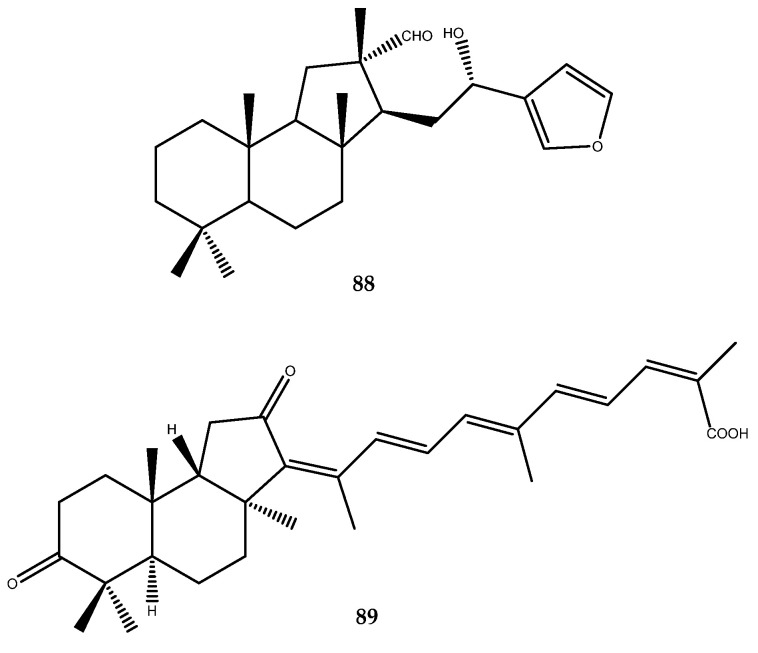
Structures of hyrtiosal (**88**) and stellettin G (**89**).

Hyrtiosal (**88**, Figure 18), which is a sesterterpenoid isolated from the Okinawan marine sponge *Hyrtios erectus,* was identified as a noncompetitive PTP1B inhibitor, with an IC_50_ value of 42.0 μM, thereby also enhancing GLUT4 membrane translocation [69]. Furthermore, Sun et al. showed that hyrtiosal displays extensive cellular effects in PI3K/AKT activation, glucose transport, and TGFb/Smad2 signaling.

An isomalabaricane triterpene, stellettin G (**89**, Figure 18), which was isolated from the Hainan sponge *Stelletta* sp., showed a strong inhibitory activity against PTP1B in displaying an IC_50_ value of 4.1 ± 0.9 μM [70].

#### 3.1.2. Sterols

**Figure 19:**
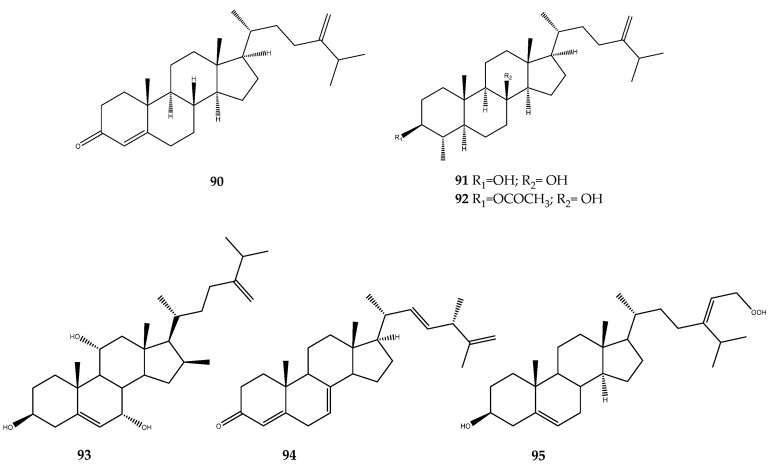
Structures of sterols **90**–**95**.

Chemical investigation of the soft coral *Sinularia depressa* resulted in the isolation of several steroids. Among these, compounds **90** and **91**, as well as its monoacetylated derivative **92** (Figure 19), exhibited potent inhibitory activity, with IC_50_ values of 15.3, 19.5, and 22.7 μM, respectively, against PTP1B [71].

Several polyhydroxylated steroids have been isolated from the South China Sea soft corals *Sarcophyton trocheliophorum* and *Sinularia flexibilis*, and, in particular, 7α-hydroxy-crassarosterol A (**93**, Figure 19) displayed a weak PTP1B inhibitory activity, with an IC_50_ value of 33.05 μM [72]. The marine sponge *Xestospongia testudinaria* was found to be a rich source of marine sterols; some of them were endowed with PTP1B inhibitory activity. In particular, compound **94** (Figure 19) showed an IC_50_ value of 4.27 ± 0.55 μM [73]. Uncommon sterols possessing unique side chains characterized by a hydroperoxyl-group were isolated from the same marine sponge. One of them, compound **95** (Figure 19), displayed an interesting inhibitory activity against PTP1B of 5.8 μg/μL [74].

#### 3.1.3. Brominated Metabolites

**Figure 20:**
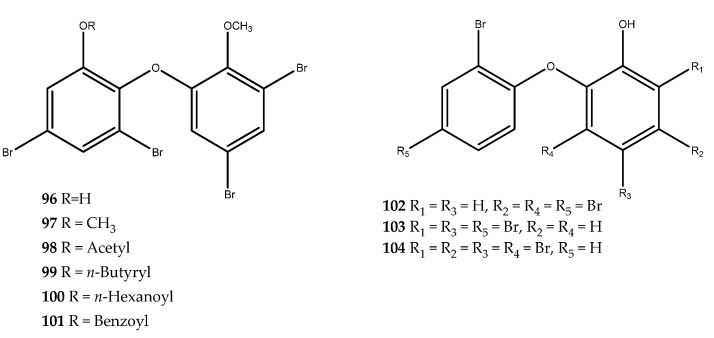
Structure of polybromodiphenyl ether derivatives **96**–**104**.

The polybromodiphenyl ether, compound **96** (IC_50_ = 0.85 μM, Figure 20), which was isolated from the Indonesian marine sponge *Lamellodysidea herbacea*, was found to be a potent PTP1B inhibitor, with an IC_50_ value in the low-micromolar range, but it resulted in cytotoxicity against two human cancer cell lines (HCT-15 and Jurkat cells). To overcome this issue, Yamazaki and coworkers operated modifications via semisynthesis to obtain the derivatives **97**–**101** (Figure 20): 3,5-dibromo-2-(3′,5′-dibromo-2′-methoxyphenoxy)-1-methoxybenzene (**97**, IC_50_ = 1.7 μM), 3,5-dibromo-2-(3′,5′ -dibromo-2′ -methoxyphenoxy)phenyl ethanoate (**98**, IC_50_ = 0.62 μM), 3,5-dibromo-2-(3′,5′-dibromo-2′-methoxyphenoxy)phenyl butanoate (**99**, IC_50_ = 0.68 μM), 3,5-Dibromo-2-(3′,5′-dibromo-2′-methoxyphenoxy)phenyl benzoate (**100**, IC_50_ = 0.69 μM), and 3,5-Dibromo-2-(3′,5′-dibromo-2′-methoxyphenoxy)phenyl benzoate (**101**, IC_50_ = 0.97 μM). The esterification of the phenol group of compound **96** increased the activity against PTP1B, but it did not reduce the cytotoxicity. Instead, the methylation moderately reduced the activity against the target; therefore, compound **97** is the most promising compound, since it showed reduced cytotoxicity [75]. From the same sponge, other polybromobiphenyl ether derivatives, 2-(2′,4′-dibromophenoxy)-3,5-dibromophenol (**102**, IC_50_ = 5.3 μM), 2-(2′,4′-dibromophenoxy)-4,6-dibromophenol (**103**, IC_50_ = 7.8 μM), and 2-(2′-dibromophenoxy)-3,4,5,6-tetrabromophenol (**104**, IC_50_ = 5.3 μM, Figure 20), were identified as PTP1B inhibitors [76].

**Figure 21:**
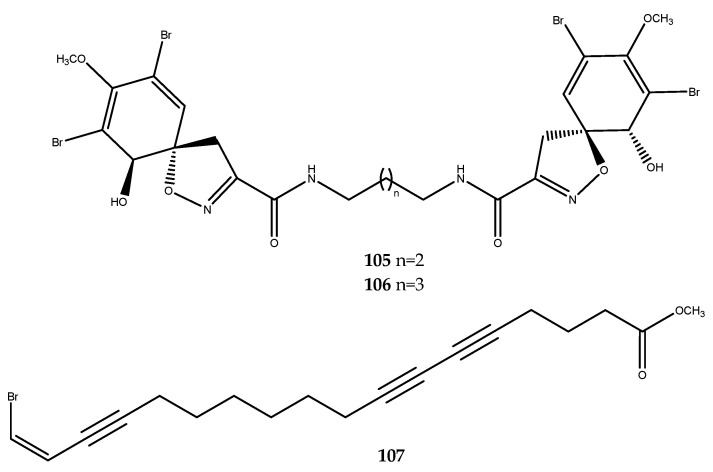
Structures of bromo-spiroalkaloids (compounds **105** and **106**) and brominated lipid (compound **107**).

Two bromo-spiroalkaloids, compounds **105** and **106** (Figure 21), were isolated from the marine sponge *Fascaplysinopsis reticulata*, which was collected from the Xisha Island; they displayed a noteworthy PTP1B inhibition activity, with IC_50_ values of 7.67 and 11.25 μM, respectively [77].

Brominated lipids were isolated from the diethyl ether soluble portion of the acetone extract of the Chinese marine sponge *Xestospongia testudinaria*, which was treated with diazomethane. Compound **107** (Figure 21) exhibited a valuable inhibitory activity against PTP1B, with an IC_50_ value of 5.30 ± 0.61 μM [78].

#### 3.1.4. Polyketides

**Figure 22:**
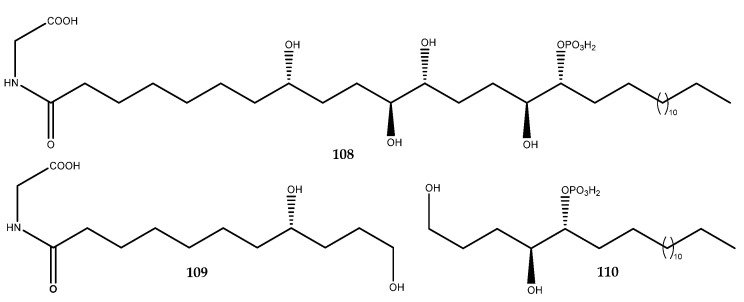
Structure of phosphoeleganin (**108**) and its semisynthetic derivatives **109** and **110**.

Few molecules belonging to this family were classified as PTP1B inhibitors, and phosphoeleganin (**108**, Figure 22) represents the most explored chemotype of this group. This monophosphorylated polyketide has been isolated from the Mediterranean ascidian *Sidnyum elegans* by our research group. The determination of its complex structure and the absolute configuration of its stereocenters, which is characterized by a long aliphatic carbon chain equipped with hydroxy groups, a phosphate moiety, and a glycine head, required an extensive spectroscopic analysis and chemical derivatizations [79,80]. Phosphoeleganin was demonstrated to inhibit not only PTP1B (IC_50_ = 1.3 ± 0.04 µM), but also aldose reductase enzyme (see paragraph 2.4) [81]. To further explore and outline some structure activity relationships, in our previous work, we chemically manipulated the natural metabolite via its oxidative cleavage, thereby leading to the obtainment of fragments **109** and **110** (Figure 22).

Preliminary screening of the two fragments (compounds **109** and **110**) on PTP1B inhibition showed that compound **109** was inactive on the enzyme, while compound **110** retained the inhibitory activity for the enzyme in displaying an IC_50_ value of 6.7 ± 3.3 μM, thus suggesting that the phosphate group was necessary to counteract the enzyme counterpart, as has been supported by docking analyses [82]. However, the higher increase in the IC_50_ value than the parent compound **110** also suggests the relevance of the whole structure for the stronger inhibition of PTP1B, which is probably due to the presence of the amino acid head or to the greater length and flexibility of the carbon chain. Moreover, synthetic simplified analogues were prepared and screened, which not only confirmed the importance of the phosphate group for the inhibition of PTP1B, but also its correct orientation was ensured by the absolute stereochemistry of the 1,2-monophosphorylated system [82]. This extended study confirmed phosphoeleganin and, thus, the polyketide scaffold as a suitable chemotype for the development of new antidiabetic leads.

**Figure 23:**
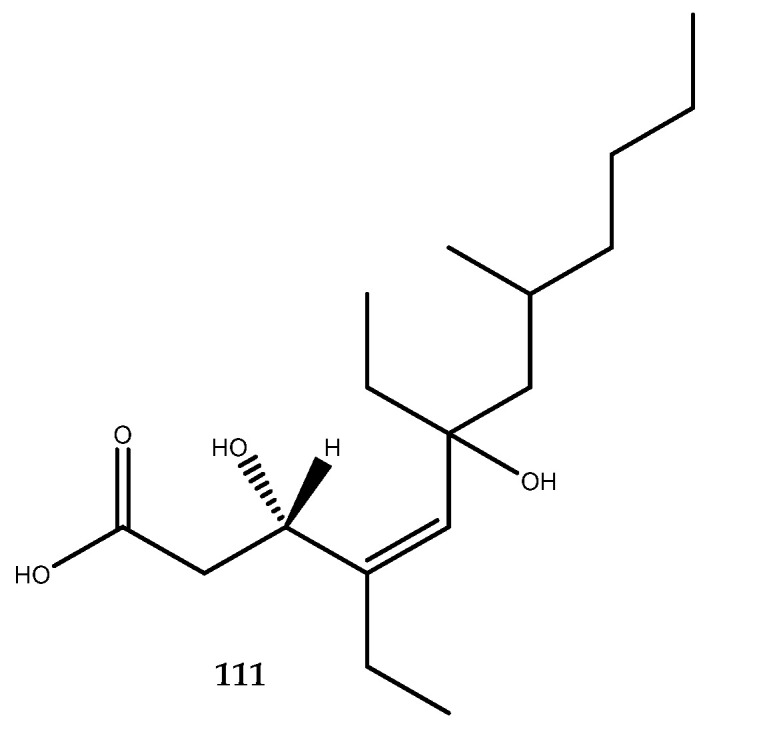
Structure of woodylide C (**111**).

Some polyketides named woodylides were isolated from the ethanol extract of the South China Sea sponge *Plakortis simplex*. One of them, woodylide C (**111**, Figure 23) expressed an interesting inhibitory activity against PTP1B, with an IC_50_ of 4.7 µg/mL. However, no further studies were performed to identify the pharmacophore of this molecule [83].

#### 3.1.5. Miscellaneous Compounds

**Figure 24:**
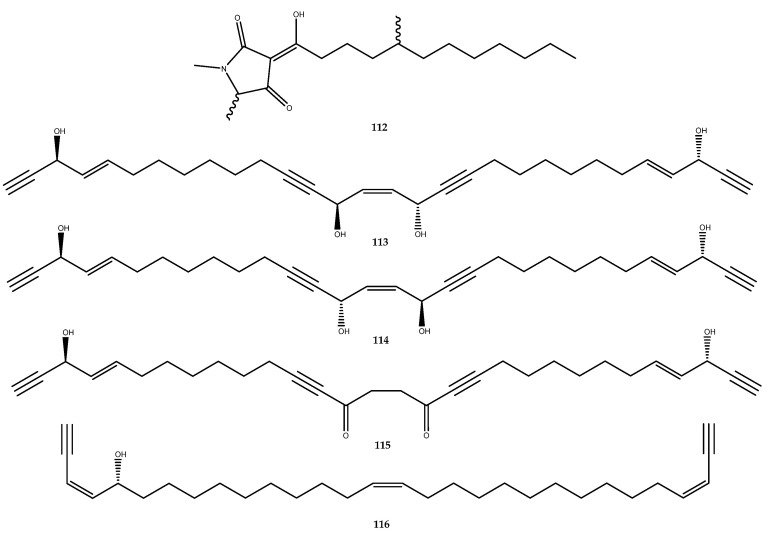
Structures of melophlin C (**112**) and some active polyacetylene derivatives **113**–**116**.

Melophlins are a family of molecules that are tetramic acid derivatives with a long alkyl chain. Among these, melophlin C (**112**, Figure 24), which was isolated from the Indonesian marine sponge *Petrosia* sp., was the first molecule of this class to be assessed for its inhibitory activity against PTP1B, thereby revealing an IC_50_ value of 14.6 μM [84,85].

The bioassay-guided separation of the sponge *Halichondria* cf. *panicea* led to the isolation of the polyacetylene compounds **113**–**116** (Figure 24), and, for the first time, the inhibitory effect on PTP1B of this class of molecules was investigated. Petrosynol (**114**, 28.9 ± 4.5% inhibition at 21.6 µM) was less potent than its diastereoisomer, isopetrosynol (**113**, IC_50_ = 8.2 ± 0.3 μM). Therefore, the configurations of OH groups at C-14 and C-17 markedly affect this activity. Furthermore, compounds **115** and **116** were also found to be PTP1B inhibitors in showing IC_50_ values of 7.8 ± 0.5 and 12.2 ± 0.5 μM, respectively [86].

**Figure 25:**
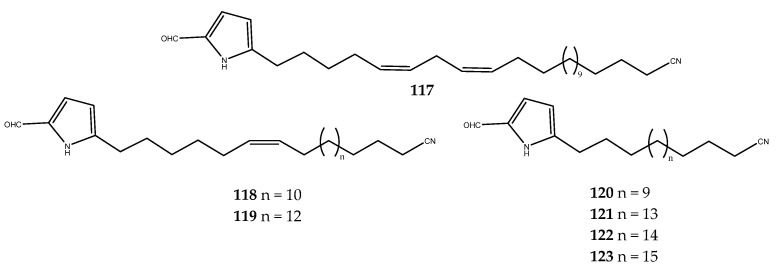
Structures of 5-alkylpyrrole-2-carboxaldehyde derivatives **117**–**123**.

Some 5-alkylpyrrole-2-carboxaldehyde derivatives, **117**–**123** (Figure 25), which are named mycalenitriles, were isolated from the South China Sea sponge *Mycale lissochela* and assayed for their inhibitory activity against PTP1B. All of the isolated compounds, except for compound **120**, exhibited PTP1B inhibitory activities (**117**, IC_50_ = 8.6 ± 0.9 μM; **118**, IC_50_ = 10.0 ± 0.2 μM; **119**, IC_50_ = 3.1 ± 0.1 μM; **121**, IC_50_ = 26.2 ± 2.1 μM; **122**, IC_50_ = 28.2 ± 3.1 μM; and **123**, IC_50_ = 12.5 ± 1.4 μM). Based on the IC_50_ values, all of the 5-alkylpyrrole derivatives with an unsaturated side chain (compounds **117**–**119**) were more active than the compounds **120**–**123**, thereby indicating that the unsaturated aliphatic side chain may be useful for the pharmacological activity against PTP1B [87].

### 3.2. α-Glucosidase Inhibitors

The enzyme α-glucosidase is a carbolytic enzyme that catalyzes the liberation of D-glucose from the non-reducing end of starch and disaccarides. The inhibition of this enzyme retards the absorption of glucose after meals, thus decreasing postprandial hyperglycemia [88].

**Figure 26:**
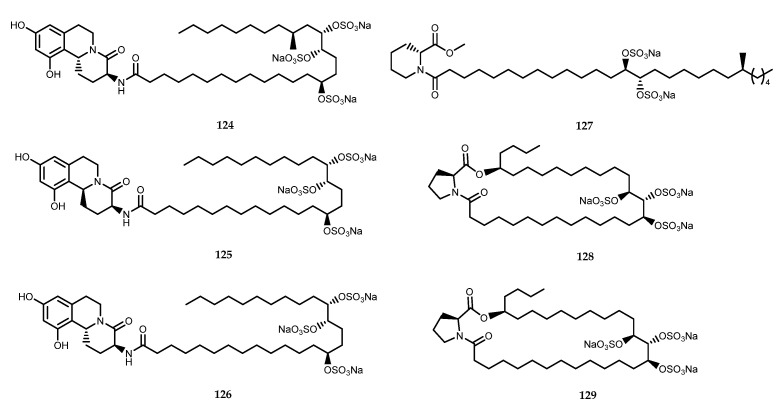
Structures of sulfates isolated from *Penares* sp. (**124**–**129**).

The chemical investigation of the metabolic content of marine sponges belonging to the genus *Penares* by the Kanao research group led to the identification of trisulfate compounds with potent α-glucosidase inhibitory activity. Schulzeines A–C (compounds **124**–**126**, Figure 26), which were isolated from the marine sponge *Penares schulzei*, are three isoquinoline-derived alkaloids that are characterized by a long fatty acid chain equipped with a trisulfate moiety, which resulted in them being potent α-glucosidase inhibitors, with IC_50_ values within 48–170 nM. [89]. Penasulfate A (**127**, Figure 26), which was isolated from a marine sponge *Penares* sp., showed a D-pipecolic acid-derived structure, a disulfate moiety, and an inhibitory activity against α-glucosidase, with an IC_50_ value of 3.5 mg/mL [90]. Penarolide sulfates A_1_ and A_2_ (**128**–**129**, Figure 26), which were isolated from the same sponge genus, *Penares* sp., shared the same scaffold, which consists of proline–macrolide trisulfates. Compounds **128** and **129** inhibited α-glucosidase, with IC_50_ values of 1.2 and 1.5 mg/mL, respectively [90,91].

**Figure 27:**
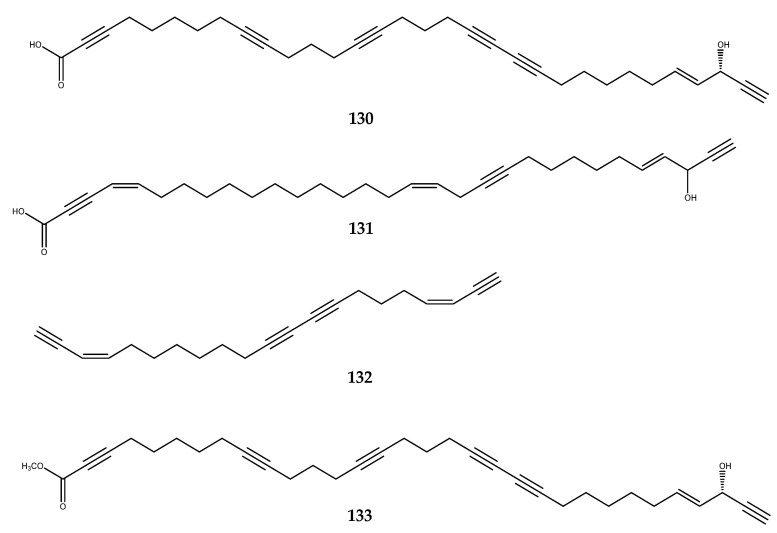
Structures of polyacetylenic acids from marine sponges (**130**–**133**).

Polyacetylenic acids are a wide group of secondary metabolites of marine sponge origin with several biological effects. Among them, callyspongynic acid (**130**), a C_32_ polyacetylenic acid isolated from the Japanese sponge *Callyspongia runcate*, and corticatic acid (compound **131**, Figure 27), a C_31_ polyacetylenic acid isolated from the sponge *Pellina triangulate*, inhibited α-glucosidase, with IC_50_ values of 0.25 µg/mL and 0.16 µg/mL, respectively [92,93]. Petrosynol (**114**) was also reported for its inhibitory effect against α-glucosidase, with an IC_50_ value of 4.08 µg/mL [92]. On the other hand, the polyacetylene hydrocarbons callytetrayne (**132**) and methyl callyspongynate (**133**, Figure 27) [94] were completely inactive, thereby highlighting the key role exerted by both carboxylic acid and allylic alcohol being linked to an acetylene for the inhibition of this target [92].

**Figure 28:**
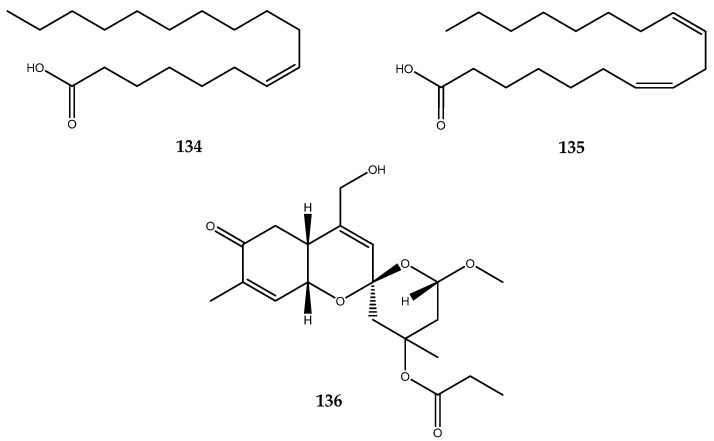
Structures of unsaturated fatty acids **134** and **135**, as well as the tricyclic spiroketal compound **136**.

The 7-(Z)-octadecenoic acid (**134**) and 7-(Z)-10-(Z)-octadecadienoic acid (compound **135**, Figure 28), which are two unsaturated fatty acids, were obtained from the body wall of *Stichopus japonicus*. The IC_50_ values of compounds **134** and **135** against *S. cerevisiae* α-glucosidase were 0.51 and 0.49 μg/mL, respectively, which were lower than the 0.67 and 0.60 μg/mL values, respectively, against *B. stearothermophilus* α-glucosidase [95].

Orhan and colleagues evaluated the in vitro antidiabetic effect of the methanol extracts of some marine organisms through the evaluation of α-glucosidase and α-amylase inhibition. All the extracts were inactive on either α-glucosidase or α-amylase, except for the *D. avara* extract, which was found to be the most active on α-glucosidase. Therefore, the major compounds of *D. avara*, avarone (**19**), and avarol (**6**) were isolated and tested against α-glucosidase and, being noteworthy, they exhibited strong inhibitory activities of 86.18 ± 1.76% and 78.94 ± 1.38%, respectively, at 10 µM [96].

Clathriketal (**136**, Figure 28), a tricyclic spiroketal compound, was isolated from the Microcionidae sponge *Clathria prolifera*. Clathriketal showed significant antidiabetic properties by inhibiting α-glucosidase, with an IC_50_ value of 0.43 ± 0.02 mM. The great potential as antidiabetic agent of this molecule lies in its multitarget inhibiting activity, as it is also capable of inhibiting α-amylase and the enzyme DPP IV (see paragraphs 2.3 and 2.5) [97].

### 3.3. α-Amylase Inhibitors

The enzyme α-amylase is carbolytic enzyme that catalyzes the liberation of D-glucose from the non-reducing end of polysaccharides. The inhibition of this enzyme, as with α-glucosidase, slows the absorption of glucose, thereby reducing postprandial hyperglycemia [88].

**Figure 29:**
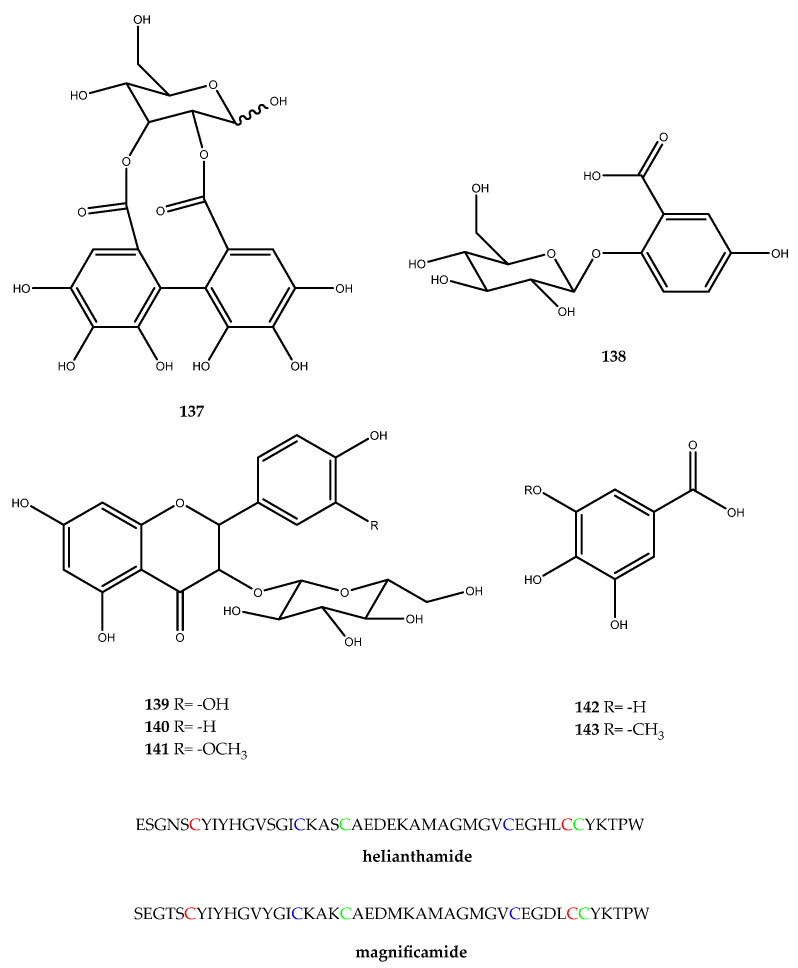
Structures of glycosides **137**–**141**, gallic acid (**142**), and its methyl ether derivative (**143**). Helianthamide and magnificamide are reported with disulfide connectivity, which is highlighted by the same color of bonding partners.

There are few examples of marine metabolites from invertebrates that express inhibitory activity against α-amylase. The bioassay0guided fractionation of the Mediterranean Sea sponge *Hemimycale collumella* led to the identification of a series of bioactive compounds: In particular, the glycosides 2,3-O-hexahydroxydiphenoyl-(α/β)-glucose (**137**) and gentisic acid 2-O-β-glucoside (**138**) were isolated from the water extract; quercetin-3-O-β-glucopyranoside (**139**), kaempferol 3-O-β-glucopyranoside (**140**), and isorhamnetin 3-O-β-glucopyranoside (**141**) were isolated from the butanol soluble material; gallic acid (**142**) was isolated from an ethyl acetate extract, and gallic acid-3-methyl ether (**143**) was isolated from a dichloromethane fraction (Figure 29). All of the isolated compounds (**137**–**143**) were tested against the α-amylase enzyme at 600 µg/mL, and all of them exerted a strong inhibition. Indeed, compound **141** was the most active in the series, with a percent of inhibition of 25.3 ± 5.8, followed by compound **139** with 32.4 ± 6.5, compound **143** (52.0 ± 8.7), compound **140** (59.2 ± 5.4), compound **138** (72.4 ± 6.2), gallic acid (76.2 ± 5.9) and, at last, compound **137**, with a percent of inhibition of 80 ± 5.1 [98].

The already described clathriketal (**136**, Figure 28), which was isolated from the Microcionidae sponge *Clathria prolifera*, was found to be a multitarget antidiabetic compound. In addition to the already reported activity against α-glucosidase, the molecule is also active against the DPP-IV enzyme and is capable of inhibiting α-amylase, with an IC_50_ value of 0.41 ± 0.03 [97].

The high-throughput screening analysis of some marine extracts led to the isolation of helianthamide from the Caribbean Sea anemone *Stichodactyla helianthus*. Helianthamide is a 44-residue peptide (4716 Da, Figure 29) and represents a strong inhibitor of human salivary α-amylase, with a constant of inhibition (Ki) of 10 pM. This molecule can be regarded as the first example of an α-amylase inhibitor with a peptide nature, thereby paving the way for the identification of new inhibitors belonging to this class [99].

Continuing on the chemical investigation of sea anemones, another α-amylase inhibitor was found in *Heteractis magnifica* mucus, magnificamide (Figure 29), which shares 84% sequence identity with the previously mentioned compound. Magnificamide (4770 Da) is a 44-residue peptide, and it was tested against both human salivary amylase and porcine pancreatic amylase, wherein it showed a Ki of 7.7 ± 1.5 nM and 0.17 ± 0.06 nM, respectively [100,101]. It is noteworthy that the advantage of these two peptides is the easy obtainment of active compounds, as it can be produced by the recombinant protein expression technique using bacterial cultures. This extreme versatility of production highlights the mentioned compounds as potential tools for the development of new peptide-like therapeutic agents for the treatment of T2DM.

### 3.4. Kinases Inhibitors

Glycogen synthase kinase-3 (GSK-3) is a serine–threonine kinase involved in the regulation of many cell functions. In particular, GSK-3 is strictly involved in the phosphorylation of the glycogen synthase enzyme. The phosphorylation event inactivates glycogen synthase, thereby inhibiting the glycogen synthesis process, and, for this reason, the inhibition of this enzyme is a therapeutic goal in type 2 diabetes mellitus. GSK-3 selective inhibition improves insulin-stimulated glucose transport activity by enhancing postinsulin receptor insulin signaling and GLUT-4 glucose transporter translocation. The current synthetic inhibitors of GSK-3 include thiadiazolidindiones, pyridyloxadiazoles, pyrazolopyrimidines, and maleimides [20].

**Figure 30:**
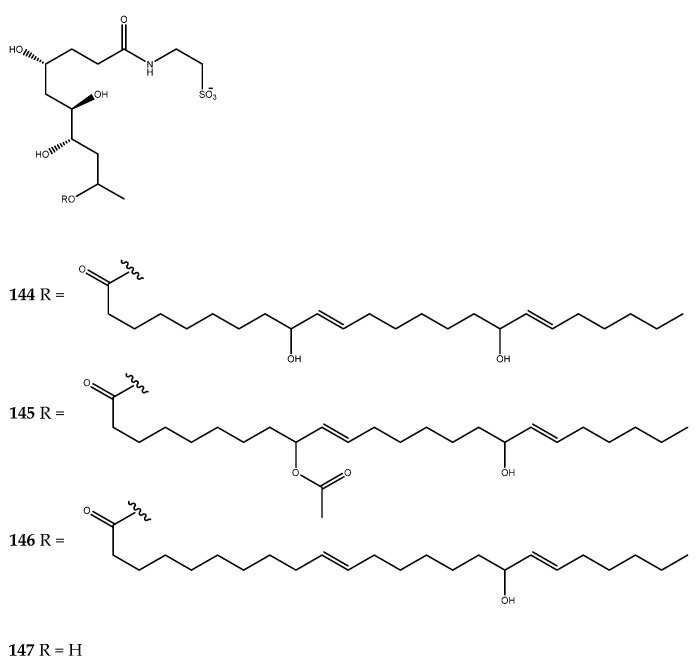
Structures of carteriosulfonic acids A–C (**144**–**146**) and the semisynthetic derivative **147**.

The carteriosulfonic acids A–C (compounds **144**–**146**, Figure 30), which were isolated from *Carteriospongia* sp. are characterized by a 4,6,7,9-tetrahydroxylated decanoic acid subunit, which is coupled through an amide bond with taurine and further esterified at O-9 with a long chain containing allylic alcohol functionality. Compounds **144**–**146** were identified as GSK-3β inhibitors in displaying IC_50_ values of 12.5, 6.8, and 6.8 µM, respectively. Desacyl-carteriosulfonic acid (**147**) was synthesized from the natural compounds to investigate the relevance of the long-chain fatty acid portion for enzyme inhibition. The loss of the pharmacological activity of compound **147** revealed that the acyl group is fundamental for the activity [102].

**Figure 31:**
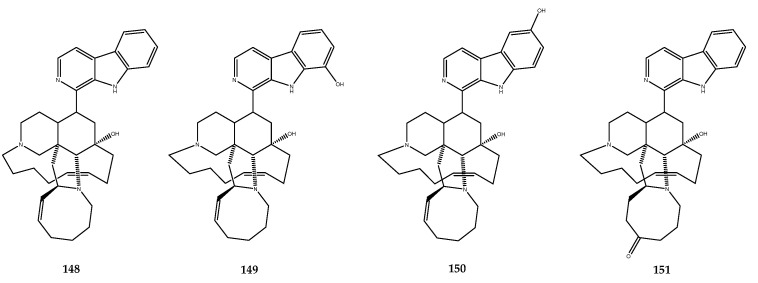
Structures of natural manzamine derivatives **148**–**151**.

Manzamines are β-carboline alkaloids that are characterized by a complex polycyclic complex, and manzamine A (**148**, Figure 31) is the first discovered molecule belonging to this class of compounds. Hamann and coworkers investigated the chemical content of the Indonesian marine sponge *Acanthostrongylophora* sp. to find new manzamine-related compounds that were capable of inhibiting GSK-3β. Several natural metabolites were isolated, and, among them, manzamine A (**148**), 8-hydroxymanzamine A (**149**), 6-hydroxymanzamine A (**150**), and manzamine E (**151**, Figure 31) were found to be inhibitors of the considered enzyme, with IC_50_ values of 10.2, 4.8, 16.6, and 25.0 μM, respectively [103].

Kinetic studies were performed to evaluate the inhibition mechanism of manzamine A, which was identified as a noncompetitive inhibitor of GSK-3β. Considering the potential of this polycyclic scaffold, Hamann and his research group started conducting activity structure relationship studies using a semisynthetic approach to identify the pharmacophore that was responsible for the pharmacological activity and to identify new modifications of the scaffold that could improve the inhibition. Both carboline and ircinal A, the chemical precursors of manzamine A, were tested against the enzyme, but they were inactive, thereby highlighting the entire manzamine moiety as an essential requirement for this activity [103]. First, the effect of the substituents on the carboline moiety was observed. Compound **149**, which presents an 8-OH group unlike in manzamine A (**148**), exerted a stronger inhibitory activity, thus highlighting the relevance of polar groups on the carboline skeleton. Some semisynthetic derivatives of compound **149**, in which the 8-OH group was replaced by OTs, OMe, OEt, and O-*i*-But groups (compounds **152**–**155**, Table 1), were prepared and screened. For all of the derivatives, the activity was comparable to the parent compound, except for the compound endowed with the isobutyl group, which led to a total loss of pharmacological activity. However, even when considering the active derivatives, the natural compound **149** was the most potent of the series.

In addition, some modifications to the aliphatic heterocyclic system were performed (**156**–**166**, Table 1). The saturation of the double bond between positions 15 and 16 led to an inactive derivative (compound **161**), while the modification in the cycloottane ring led to different activity changes. Indeed, the replacement of the double bond in the cycloottane ring with an epoxy functionality did not affect the activity in compound **158**, while the substitution with a carbonyl group in position 31 led to a less potent derivative **160**. The functionalization of the hydroxyl group with bulky arylic groups of the carbonyl derivative **160** allowed us to recover the loss activity. Moreover, the dehydration of manzamine A led to the obtainment of compound **165**, which was more potent than the parent compound. The activity of each reported compound was expressed as percent of inhibition using a concentration of 25 μM of inhibitor and, for some compounds, as the IC_50_ value (Table 1) [103].

**Figure 32:**
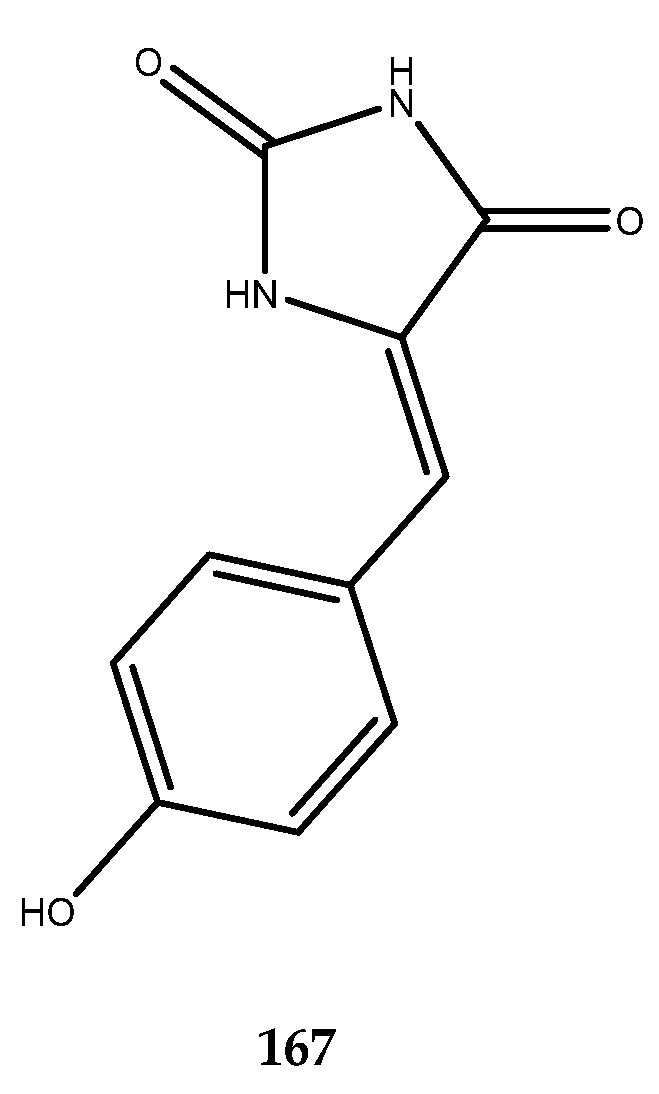
Structure of (*Z*)-5-(4-hydroxybenzylidene)hydantoin (**167**).

The chemical investigation of the Red Sea sponge *Hemimycale arabica* provided the isolation of the known (*Z*)-5-(4-hydroxybenzylidene)-hydantoin (**167**, Figure 32), which was identified as a novel GSK-3β inhibitor, with an IC_50_ value of 13.7 ± 1.2 µM. These results highlight phenylmethylene hydantoin as a new chemotype to explore in the search for novel synthetic GSK-3β inhibitors. Khanfar and colleagues developed an efficient and simple synthetic method to generate a small library of hydantoins to replace the 4-hydroxyphenyl group with other arylic and heterocyclic systems. In particular, compounds endowed with a parasubstituted phenyl group with electron donating groups such as SCH_3_, SCH_2_CH_3_, and N(CH_2_CH_3_)_2_ showed higher inhibitory activity than the natural parent compound [104].

**Figure 33:**
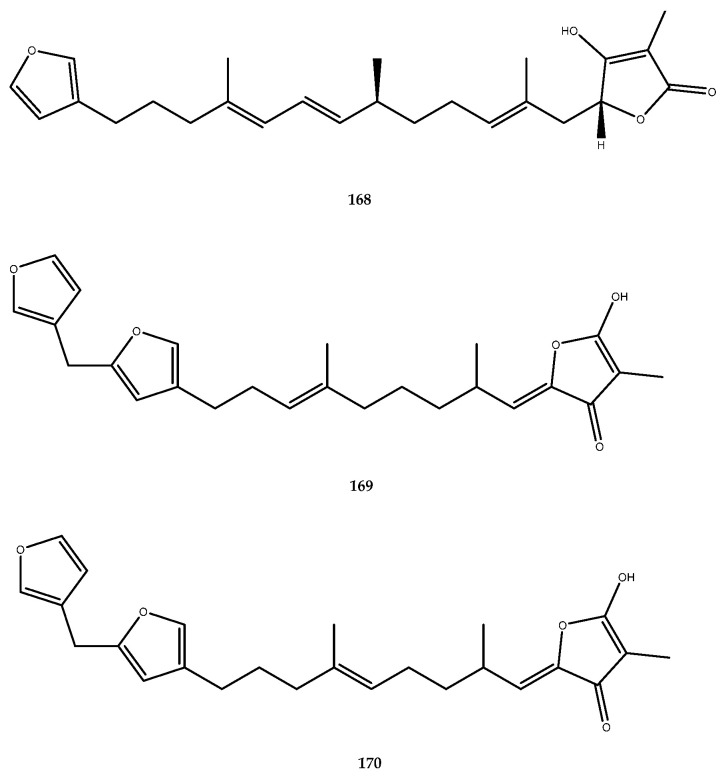
Structures of palinurin (**168**) and of ircinin-1 and ircinin-2 (**169** and **170**).

The linear furanosesquiterpene palinurin (**168**, Figure 33) was isolated from the sponge *Ircinia dendroides* and exhibited a potent GSK3-β inhibitory activity, with an IC_50_ value of 1.9 μM, through a nonATP/substrate competitive mechanism. To validate this scaffold as a promising chemotype for the development of novel synthetic inhibitors, ircinin-1 (**169**) and ircinin-2 (**170**, Figure 33) were also isolated from sponges belonging to the *Ircinia* genus, and they were assessed against this target, since they have a structural similarity with compound **168**. As expected, the inhibitory activities of ircinin-1 and ircinin-2 were very similar to those determined for palinurin, with IC_50_ values of 0.8 and 2.3 μM, respectively [105].

**Figure 34:**
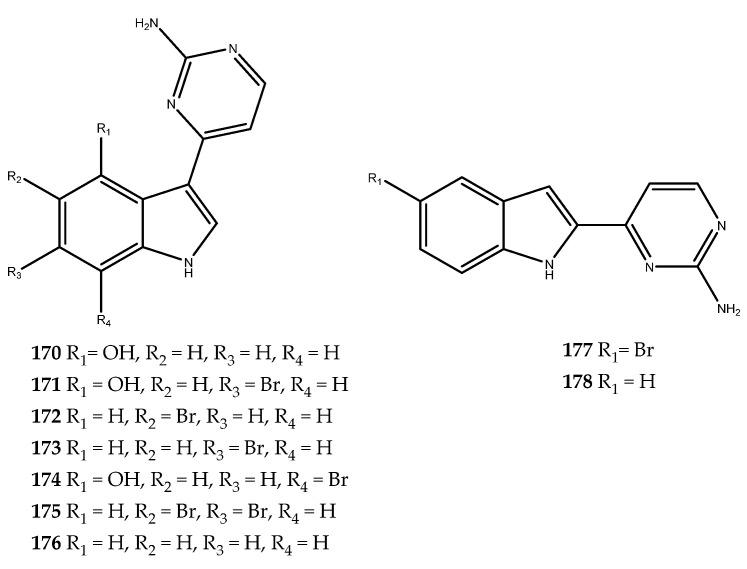
Structure of meridianins **170**–**176** and iso-meridianins **177** and **178**.

Meridianins are a family of brominated 3-(2-aminopyrimidine)-indoles, which differ based on the different substitution on the indole ring. These compounds have been isolated for the first time from the green ascidian *Aplidium meridianum*, which was collected at a depth of 100 m near to the South Georgia Islands [106]. Gompel and coworkers isolated several meridianins, compounds **170**–**178** (Figure 34), by studying specimens of *Aplidium meridianum* that were collected in the same region and screened them on a panel of kinases, including GSK-3β. The effects against this enzyme are so summarized: meridianin A (**170**, IC_50_ = 1.30 μM), meridianin B (**171**, IC_50_ = 0.50 μM), meridianin C (**172**, IC_50_ = 2.00 μM), meridianin D (**173**, IC_50_ = 2.50 μM), meridianin E (**174**, IC_50_ = 2.50 μM), meridianin F (**175**, IC_50_ = 2.0 μM), meridianin G (**176**, IC_50_ = 350.0 μM), iso-meridianin C (**177**, IC_50_ > 1000 μM), and iso-meridianin G (**178**, IC_50_ = 420.0 μM) [107,108].

Compounds **170**–**175** exhibited a similar inhibitory activity, with IC_50_ values within 0.50–2.50 μM, while compound **176** was inactive, thereby highlighting the importance of the substitution of the indole ring with bromo and/or hydroxy groups. Moreover, the structural isomers of the meridianins and iso-meridianins C and G (**177** and **178**) were inactive as well, thereby highlighting the position of the 2-aminopirimidine group as essential in the inhibition of the enzyme [108]. Han and colleagues, by applying a structural-based optimization strategy, exploited the meridianin C scaffold to generate synthetic molecules by introducing specific substituents based on the already known synthetic inhibitors of GSK-3β. The obtained compounds showed an increased potency and improved pharmacokinetics properties; thus, the meridianin scaffold has been demonstrated as a promising tool for the development of a new generation of antidiabetic drugs [109,110]. The relevance of the meridianin scaffold was also confirmed by an in silico binding study conducted by Llorach-Pares and coworkers, which aimed to identify new potential GSK-3β inhibitors among marine natural products. All of these results suggest that meridianins could be further explored to develop new therapeutic agents for the treatment of T2DM [107].

**Figure 35:**
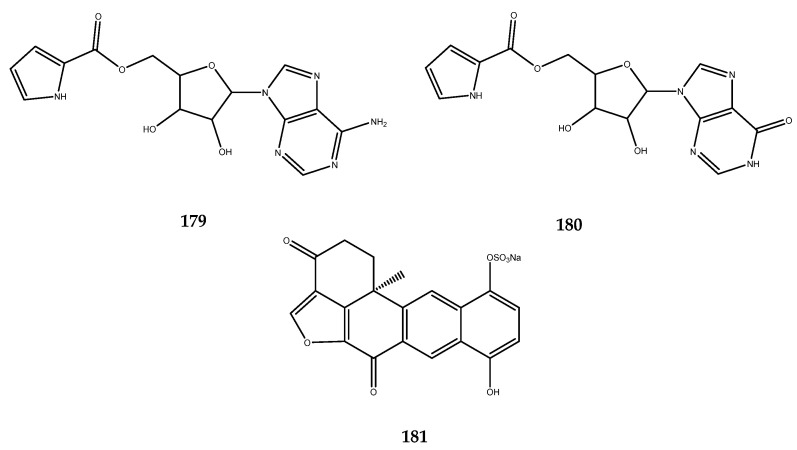
Structure of nelliellosides A and B (**179** and **180**) and the halenaquinol sulphate (compound **181**).

Nelliellosides A and B (**179** and **180**, Figure 35), which were isolated from a quadricellariid cheilostome bryozoan, *Nelliella nelliiformis*, are C-5′-substituted nucleosides that are characterized by adenine and hypoxanthine cores, respectively. These two metabolites were tested on a panel of kinases, including GSK-3β, on which they exerted a potent inhibitory activity expressed as a percent inhibition of the enzyme at a concentration of 10 μM. It was observed that compound **180** exerted 94% of enzyme inhibition, whereas compound **179** was even more active, with a complete inhibition of GSK-3β activity (100%). On this basis, the research group that isolated these molecules developed a small library of molecules in which the nitrogenous base was replaced by the guanine, as well as shifting the ester group from C2 to C3 of the pyrrole ring and retaining the three different nitrogenous bases [111]. The obtained molecules were screened with brilliant preliminary results, thereby confirming this scaffold as an excellent chemotype to investigate not only for the inhibition of the GSK-3β enzyme, but also for other kinases [111].

Another example of an interesting kinases inhibitor is halenaquinol sulphate (**181**, Figure 35). It is a polycyclic compound that was first isolated from the marine sponge *Xestospongia* sp., and it was demonstrated to exhibit a potent inhibitory activity against both GSK-3α/β, with an IC_50_ value of 0.61 μM [112].

**Figure 36:**
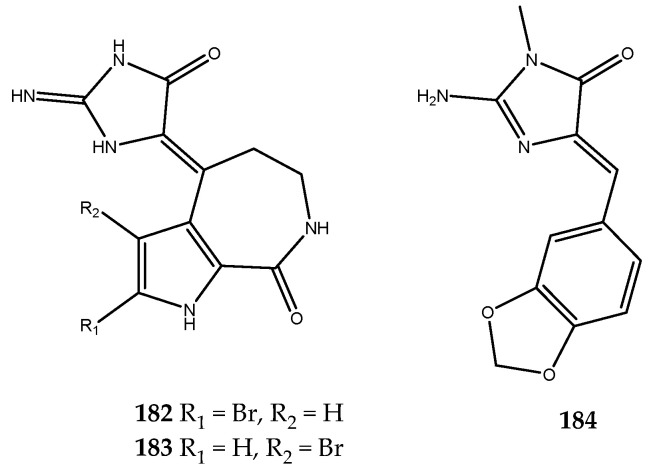
Structure of hymenialdisine (**182**), spongiacidin B (**183**), and leucettamine B (**184**).

The unspecific assay on the marine sponge *Callyspongia* sp. extract revealed inhibitory against several kinases. Thus, the bioassay-guided fractionation of the extract led to the isolation of secondary metabolites that were putatively responsible for the biological effects. Among these, GSK-3β was inhibited from hymenialdisine (**182**, Figure 36), a bromopyrrole alkaloid characterized by the 2-aminoimidazolin-4-one scaffold, which showed a strong inhibition of this enzyme, with an IC_50_ in the nanomolar range of 4.9 nM [113,114].

Analogously, spongiacidin B (**183**) and leucettamine B (**184**, Figure 36), which share the same 2-aminoimidazolin-4-one scaffold of compound **182**, were isolated for the first time from the sponges *Hymeniacidon* sp. and *Leucetta microraphis*, respectively [115,116]. Considering the main limitation of the use of marine products in pharmacological screening due to their limited amount, both compounds **183** and **184** were easily obtained through total synthesis and were tested on a panel of kinases, including GSK-3α/β. Against this enzyme, both were active, but spongiacidin B (**183**) showed a significant inhibitor effect, with an IC_50_ value in the low micromolar range (0.044 μM). In contrast, compound **184** showed an IC_50_ value of 7.2 μM [114].

**Figure 37:**
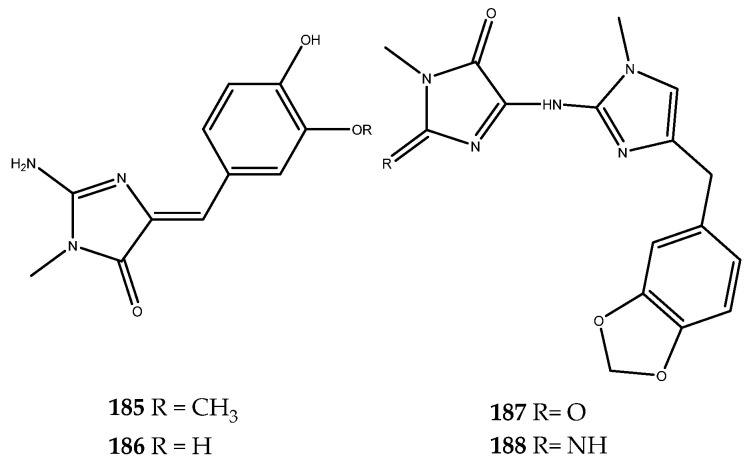
Structures of polyandrocarpamines A and B (**185** and **186**), clathridine (**187**), and clathridimine (**188**).

Promising strategies for the treatment of T2DM, as well as of of type 1 diabetes mellitus, certainly include the regeneration of pancreatic β-cells. DYRK1A kinase, a member of the DYRK family, is involved in cell growth and differentiation processes. Several evidence outcomes have shown that DYRK1A is involved in the progression of T2DM, and, therefore, inhibitors of this kinase could be important therapeutic agents for its treatment by promoting β-cell proliferation [117]. Accordingly, Loaëc and coworkers decided to investigate the effects of several marine natural products endowed with a 2-aminoimidazolin-4-one scaffold against a panel of kinases that included DYRK1A. Interestingly, all of the tested compounds were shown to be active [114]. In particular, the inhibitory effects are so summarized: polyandrocarpamines A and B (compound **185**, IC_50_ = 0.27 μM and compound **186**, IC_50_ = 0.47 μM), which were isolated for the first time from the Fijian ascidian *Polyandrocarpa* sp. [118], hymenialdisine (compound **182**, IC_50_ = 0.0033 μM), spongiacidin B (compound **183**, IC_50_ = 0.78 μM), leucettamine B (compound **184**, IC_50_ = 0.42 μM, Figure 36) [113], and clathridine (compound **187**, IC_50_ = 7.8 μM), as well as its analogue clathridimine (compound **188**, IC_50_ = 5.2 μM), which was first reported from the calcareous sponge *Clathrina clathrus* (Figure 37). Although many of these compounds exert a nonspecific inhibitory effect, as they also inhibited other kinases in the panel used, these results have paved the way to consider the 2-aminoimidazolin-4-one scaffold as a promising chemotype that could be further explored for the synthesis of more selective DYRK1A kinase analogues [119].

### 3.5. AR Inhibitors

Aldose reductase (AR) is a NADPH-dependent enzyme belonging to the aldo–keto reductase superfamily, and it catalyzes the first reaction of the polyol pathway, which involves the conversion of D-glucose to D-sorbitol. During normal glycemic conditions, most of glucose is phosphorylated by hexokinase, which is the first enzyme of the glycolysis pathway. Under hyperglycemic conditions, glucose is funneled through the polyol pathway, thereby increasing the intracellular sorbitol concentration. The high accumulation of sorbitol, together with other metabolites, and the increased oxidative stress cause diabetic complications such as retinopathy, nephropathy, angiopathy, and cataracts [23,120].

**Figure 38:**
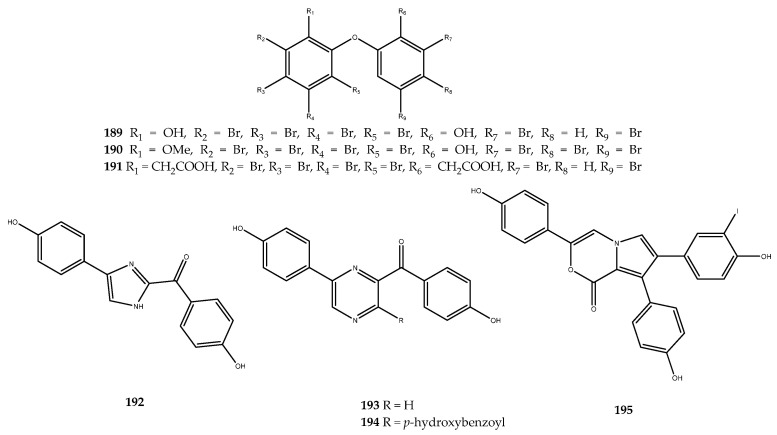
Structures of polybrominated diphenyl ethers (compounds **189**–**191**) and alkaloids **192**–**195**.

A potent inhibition of AR has been observed by testing the marine polybrominated diphenyl ether compound **189** (Figure 38), which was isolated from the marine sponge *Dysidea herbacea,* which showed an IC_50_ value of 6.4 ± 1.1 μM [121]. It is worthy to be noted that compound **189** showed a similar IC_50_ to that of the well-known orally active AR inhibitor sorbinil (IC_50_ = 3.6 μM) [122].

Thus, a cluster of polyhalogenated analogues were obtained from semisynthesis using compound **189** as building block and were screened to investigate the structure–activity relationships [123]. In this review, we only underlined the effects of the active compounds in the series, which were compounds **190** and **191** (Figure 38), with IC_50_ values of 5.5 ± 1.4 and 25.0 ± 0.1 μM, respectively. Indeed, it was observed that, by modifying both hydroxyl groups, the AR inhibitory effects were complete, except for compound **191**. In this derivative, despite the replacement of the -OH groups with a free carboxylic function, the activity was retained but with a significant decrease in the efficacy, thereby confirming the importance of free hydroxy groups in causing a decrease in the inhibitory activity [123].

Other examples of phenolic derivatives characterized by the presence of heterocyclic systems in the structure have been identified as AR inhibitors. Among these, the imidazole- and pyrazine-derived alkaloids **192**, **193**, and **194** (Figure 38) were isolated from the red ascidian *Botryllus leachii*, and they showed moderate AR inhibition, with IC_50_ values of 21.4, 41.4, and 19.4 μM. In contrast, lukianol B (**195**, Figure 38) [124], an alkaloid that is characterized by an *N*-alkylpyrrole-2-carboxilic acid moiety linked to two 4-hydroxyphenyl groups and a 3-iodo-4-hydroxyphenyl group, was isolated from an unidentified tunicate and displayed an IC_50_ value of 0.6 μM against AR, which was more potent than sorbinil by a 6-fold magnitude [125].

**Figure 39:**
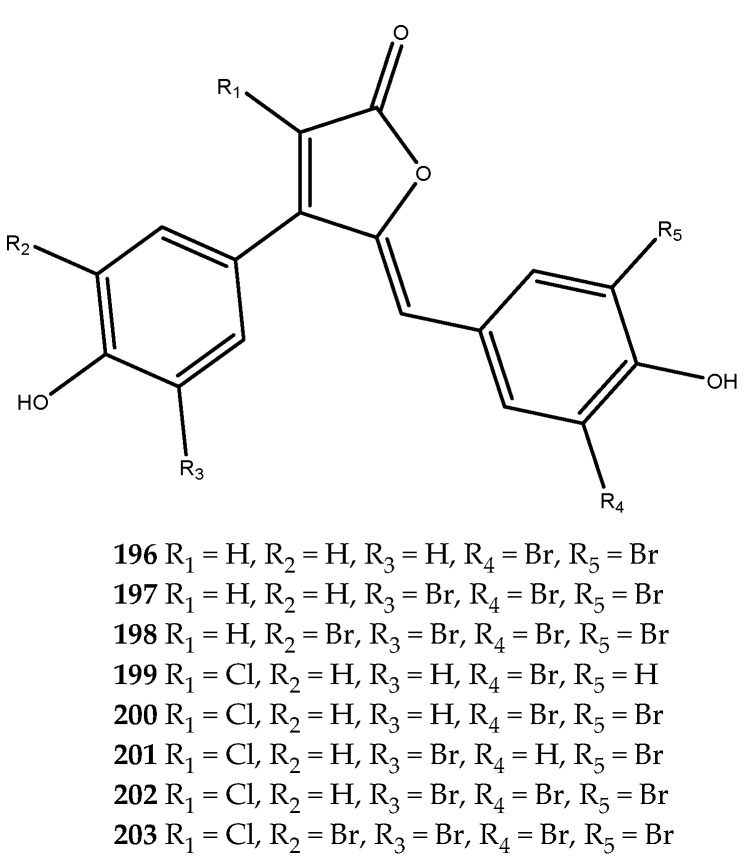
Structure of rubrolides **196**–**203**.

Phenolic-derived marine natural products linked to a γ-lactone ring are known as rubrolides. These metabolites and their synthetic analogues are endowed with a wide range of bioactivities such as antibiotic, anti-inflammatory, cytotoxic, and antifouling [28,126,127]. Moreover, a series of naturally occurring rubrolides isolated from the ascidians *Ritterella rubra* and *Synoicum blochmanni* have also been tested against the AR enzyme, and many of them were active: this includes compound **196** (IC_50_ > 57 μM), compound **197** (IC_50_ = 48.1 μM), compound **198** (IC_50_ = 19.8 μM), compound **199** (IC_50_ = 46.2 μM), compound **200** (IC_50_ = 0.8 μM), compound **201** (IC_50_ = 16.9 μM), compound **202** (IC_50_ = 12.7 μM), and compound **203** (IC_50_ = 18.7 μM, Figure 39). Among all of the screened rubrolides, compound **198** was identified as the most potent inhibitor in displaying the lowest IC_50_, thus highlighting that the replacement of the hydrogen at the α-carbon (R_1_) with a chlorine atom on the γ-lactone ring strongly affected the activity with respect to compound **196**, which was completely inactive [125].

**Figure 40:**
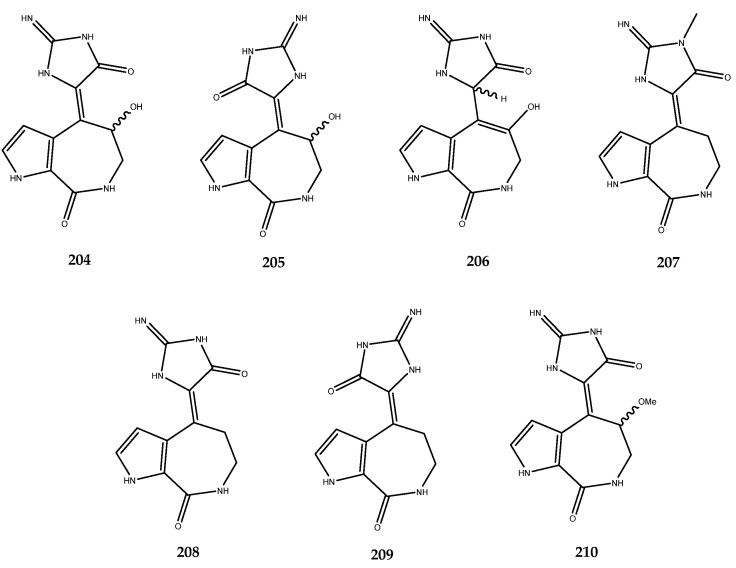
Structures of spongiacidins **204**–**210**.

Some 5/7/5-tricyclic pyrrole-based alkaloids known as spongiacidins (Figure 40) were isolated from the Xisha Islands sponge *Stylissa massa* and exerted good inhibitory activity against AR with the following IC_50_ values: compound **204** (IC_50_ = 10.0 μM), compound **205** (IC_50_ = 8.6 μM), compound **206** (IC_50_ = 12.1 μM), compound **207** (IC_50_ = 8.6 μM), compound **208** (IC_50_ = 12.0 μM), compound **209** (IC_50_ = 13.1 μM), and compound **210** (IC_50_ = 13.6 μM). SAR studies allowed for the observation that the 9-OH and 13-NMe groups may enhance the AR inhibitory activities of this spongiacidin–alkaloids family [128].

The time length of clinical trials, which often result in a reduced efficacy of the putative drugs, make it necessary to search for new and potent aldose inhibitors. For this reason, our research group is involved in the study of the metabolic content of marine invertebrates to identify new AR inhibitors and/or multitarget agents. Accordingly, the already mentioned phosphorylated polyketide phosphoeleganin (**108**, Figure 22) was identified as a dual-type inhibitor of PTP1B and AR, with an IC_50_ value of 1.3 ± 0.04 µM and IC_50_ = 28.7 ± 1.1 µM, respectively. Interestingly, phosphoeleganin represents the first example of a marine polyketide that is active against AR, against which it exerts a mixed-type inhibitory mechanism [81]. The oxidative cleavage performed on the natural compound led to the formation of fragments **109** and **110** (Figure 22), which were screened against this target. Pharmacological results revealed that both of the fragments were inactive on the AR enzyme, thereby outlining the entire structure of the polyketide as essential for the activity [82].

The chemical analysis of the Aegean sponge *D. avara* led to the isolation of the well-known sesquiterpene hydroquinone avarol (**6**, Figure 2), its oxidized form avarone (**19**), and two methyl amino derivatives, 3-(methylamino)avarone (**20**) and 4-(methylamino)avarone (**21**, Figure 3). Compounds **6** and **19**–**21** were tested against AR inhibition and were active, with IC_50_ values of 0.52 ± 0.19, 0.078 ± 0.017, 73 ± 15, and 62 ± 8 μM, respectively. The pharmacological screening demonstrated that avarone (**19**) was the most potent of the series and that it acts as a dual-type inhibitor, since it was also active on PTP1B (IC_50_ = 6.7 ± 0.6 μM). Moreover, further pharmacological characterization of **19** was carried out, thus highlighting that this compound acted as a noncompetitive mixed-type inhibitor of AR [41].

### 3.6. DPP-IV Inhibitors

Dipeptidyl peptidase IV (DPP-IV) is an exopeptidase that selectively cleaves N-terminal dipeptides from a variety of substrates, including incretin hormones glucagon-such as peptide-1 (GLP-1) and glucose-dependent insulinotropic polypeptide (GIP). Those incretines are major regulators of postprandial insulin secretion, which maintain glucose homeostasis by increasing insulin secretion and decreasing glucagon degradation [129].

To our knowledge, just one example of marine natural products capable of inhibiting DPP-IV has been reported. The activity of clathriketal (**136**, Figure 28), which was isolated from the the Microcionidae sponge *Clathria prolifera*, has already been extensively discussed in this review because of its multitarget behavior, as this molecule is an inhibitor of the carbolytic enzymes α-glucosidase (IC_50_ = 0.43 ± 0.02 mM) and α-amylase (IC_50_ = 0.41 ± 0.03 mM). To complete the profile of its antidiabetic activity, clathriketal was also confirmed as an inhibitor of the DPP-IV enzyme, with an IC_50_ value of 0.37 ± 0.03 mM [97].

### 3.7. PPAR Agonists

The peroxisome proliferator-activated receptors (PPARs) are nuclear fatty acid receptors, which play an important role in metabolic diseases such as insulin resistance, obesity, and coronary artery disease. Two subtypes of PPAR, PPARα and PPARγ, are strictly involved in the onset of T2DM. PPARγ increases glucose uptake, lipid uptake, and glucose oxidation, as well as decreases insulin resistance and free fatty acid concentration. The synergic activation of PPARα and PPARγ promotes both glucose and lipid homeostasis, as well as insulin sensitivity, and it decreases inflammation events [130].

**Figure 41:**
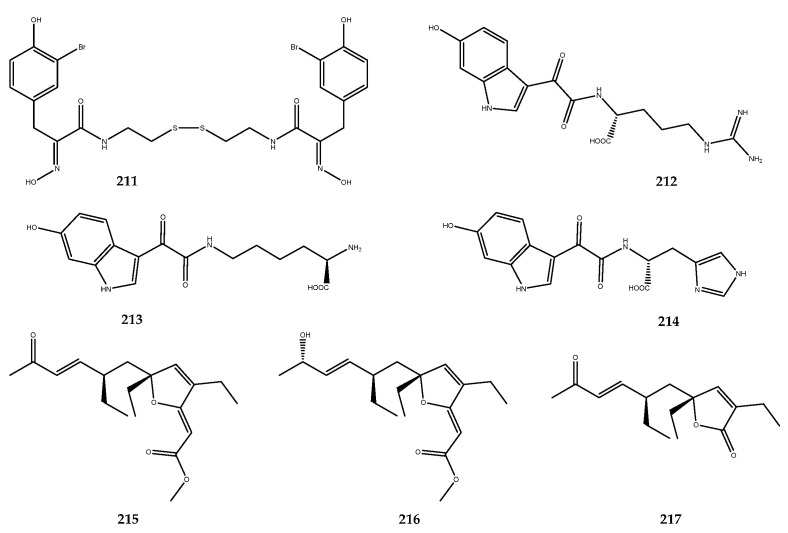
Structures of psammaplin A (**211**), compounds **212**–**214**, gracilioethers B and C (**215**–**216**), and plakilactone C (**217**).

Bioassay-guided fractionation of the sponge *Pseudoceratina rhax* led to the isolation of psammaplin A (**211**, Figure 41), which was identified as a PPARγ agonist in the MCF-7 cellular line with an EC_50_ of 5.7 µM [131]. Molecular docking studies suggested that the natural model may interact with binding sites within the PPARγ ligand binding pocket [132].

The methanol extract of the marine ascidian *Herdmania momus* was investigated and afforded several amino acid derivatives with an indole-based motif. In particular, when investigated as a PPARγ agonist, compound **212** and herdmanines I and K (**213** and **214**, Figure 41) appeared particularly interesting, since they activated the PPARγ receptor when assessed by a luciferase assay at 1 and 10 μg/mL concentrations, wherein they had higher potencies than the synthetic agonist rosiglitazone [133].

Sponges of the genera *Plakortis* and *Plakinastrella* are known to produce a great variety of oxygenated polyketides, which are obtained by combining acetyl-, propionyl-, and/or butyryl-CoA units. These metabolites belong to the plakortolide, plakinic acid, plakortic acid, plakortone, or plakortide families [134]. The bioprospecting of the marine sponge *Plakinastrella mamillaris* led to the identification of the furanylidene polyketide gracilioethers B and C (**215** and **216**), as well as plakilactone C (**217**, Figure 41), which were found to be PPARγ ligands. The transactivation assays of these compounds outlined EC_50_ values of 5, 10, and 2 µM, respectively. Compounds **215** and **217** were demonstrated to bind covalently the PPARγ ligand binding domain via a Michael addition reaction involving a cysteine residue (Cys285) and the α,β-unsaturated ketone functionality. On the other hand, compound **216**, lacking of the α,β-unsaturated ketone moiety, acted as a noncovalent agonist for PPARγ [135].

**Figure 42:**
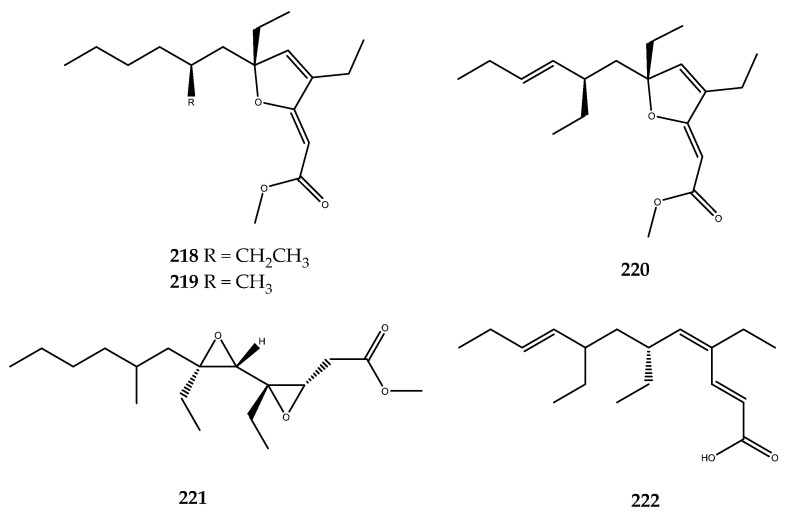
Structures of compounds **218**–**222**.

Further furanylidene-type polyketides were isolated from the Chinese sponge *Plakortis simplex*, and, among them, compounds **218**–**222** (Figure 42) were identified as selective PPARγ agonists in exerting a 2-fold induction at 50 μM. Compounds **218**–**220** may act as Michael acceptors, while compound **221** is endowed with two reactive epoxide rings as electrophile sites; therefore, these agonists could act as covalent ligands of PPARγ [136]. Compound **222**, in contrast, was the only metabolite found to be a potent dual agonist of both PPARα and PPARγ (50 μM= 2.13-fold induction; 25 μM= 1.85-fold induction; 12.5 μM= 1.42-fold induction), and its activity represents a great potential for the treatment of metabolic disorders. Taken together, these results confirm the furanylidene acetate scaffold as a possible chemotype to be explored for the development of new potential covalent agonists of PPARγ.

**Figure 43:**
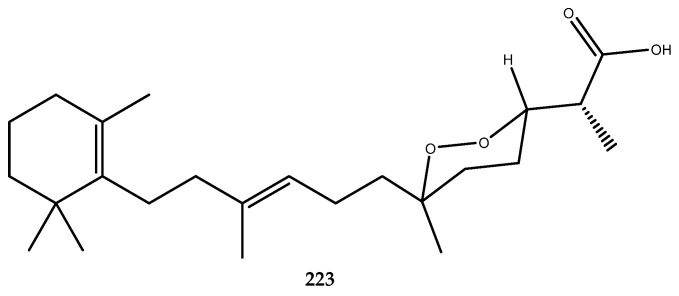
Structure of (−)-muqubilin A (**223**).

By employing a virtual screening approach of marine compounds to discover new active agents against nuclear receptors, (−)-muqubilin A (**223**, Figure 43), a cyclic peroxide norterpene, has been isolated from several sponges of the genera *Prianos* and *Diacarnus*, which were collected in the Red Sea, and it has been identified as a multitarget lead [137,138]. Pharmacological investigation highlighted this compound as a potent agonist of both PPARα and PPARγ in the low-micromolar range (1–10 µM), as well as of the retinoid X receptor (RXRα) [137].

## 4. Conclusions

**Figure 44:**
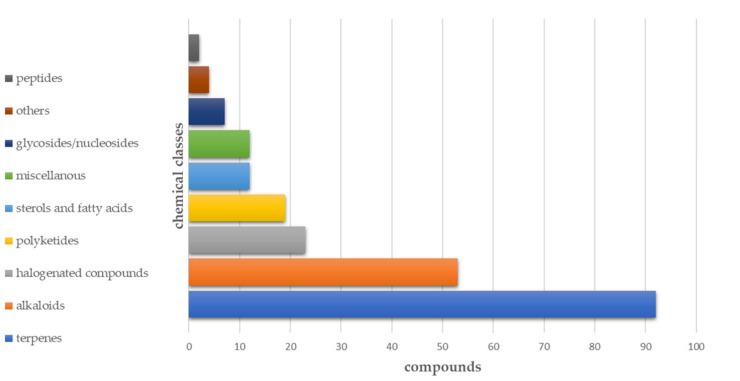
Graphical description of the main classes of MNPs and MNP-derived compounds involved in T2DM and its complications.

This review listed an array of more than 200 compounds that were isolated from marine invertebrates or obtained via semisynthetic procedures, which have been highlighted for their high antidiabetic potential. We provided an overview on the great chemical diversity (Figure 44) of marine natural products as a key tool that can advance understanding in the T2DM research field, as well as in antidiabetic drug discovery.

**Figure 45:**
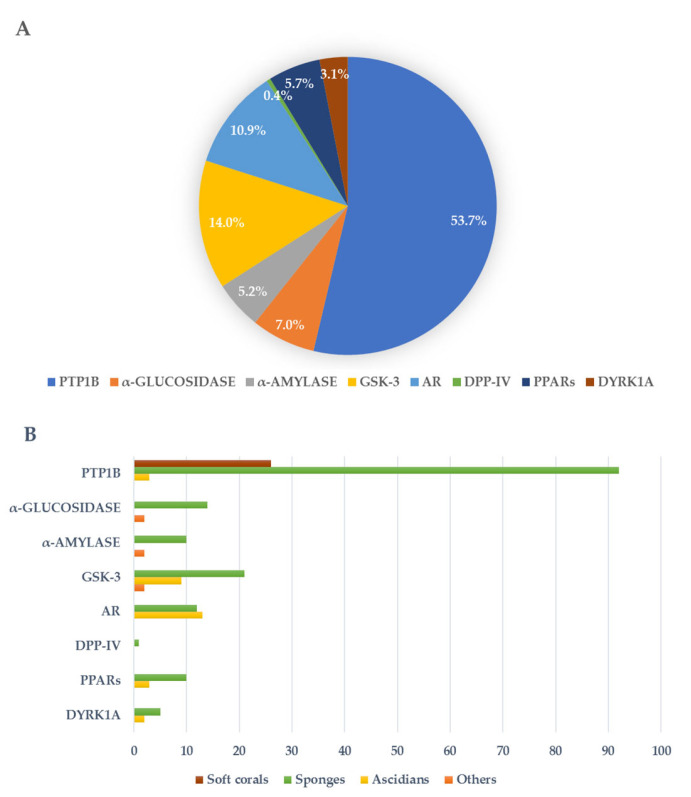
Numerical representation of the reported MNPs and their semisynthetic derivatives involved in T2DM and related complications. (**A**) Relative percentage of bioactive MNPs and MNP-derived compounds with respect to the selected targets. (**B**) Histogram of MNPs active on a specific target, categorized by the most representative invertebrates.

As explained in Figure 45A,B, the most of reported MNPs and MNP-derived compounds have been demonstrated to act as PTP1B inhibitors. Great interest has been shown toward the identification of new α-glucosidase and α-amylase inhibitors. Furthermore, to our knowledge, only one example of a DPP-IV inhibitor from marine invertebrates has been reported up to now. Interestingly, sponges yielded the most prolific source of bioactive metabolites and/or guiding chemical scaffolds (Figure 45B).

Although the marine world is evolving as a remarkable source of antidiabetic agents, so far, the therapeutic benefits in the context of T2DM have been demonstrated in vivo for a limited number of compounds. Among these, most are compounds derived from macroalgae, such as fucosterol and phlorotannins, or algal extracts, which are natural matrices that are already known as valuable nutraceuticals [139]. Among the invertebrate-derived bioactive compounds with antidiabetic potential, the most advanced compound is the PTP1B inhibitor dysidine (**2**), which is currently in preclinical trials for the treatment of diabetes [140]. It is hoped that this will give new input to the search for novel marine antidiabetic medicines, since the available data show interesting perspectives. The discussed effects of different classes of invertebrates-derived NPs with different mechanisms provide, indeed, crucial insights into the huge unexplored marine-based antidiabetic potential. Although more efforts are required to meet the upcoming challenges of the clinical utility, it is evident that a variety of promising bioactive scaffolds are available. They could lead to high level of innovation for improving antidiabetic therapeutic strategies, as well as provide new chemical platforms to be exploited by synthetic approaches following SAR analysis.

Moreover, the results reviewed in this paper support and encourage further fundamental application studies, such as testing MNPs, whose involvement in diabetes targets has already been reported, on new key enzymes with the aim to design multiple ligands that can overcome the drawbacks of polypharmacology. Thus, the future research focus could be on the development of a variety of multienzyme collaborative screening approaches, as well as the application of a variety of target enzymes, to screen and analyze multiple components simultaneously.

## Figures and Tables

**Table 1:** Percent inhibition and IC_50_ of manzamine-derived compounds **152**–**166**.

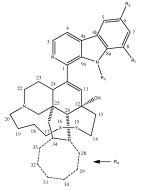							
Compound	R_1_	R_2_	R_3_	R_4_	X-Y	GSK3β Inhibition at 25 µM	GSK3β IC_50_
**152**	OTs	H	H	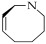	HC=CH	80.4	n.d. ^a^
**153**	OMe	H	Me	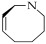	HC=CH	72.4	n.d. ^a^
**154**	OEt	H	Et	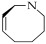	HC=CH	78.0	10.4
**155**	O-*i*-But	H	*i*-But	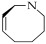	HC=CH	24.2	n.d. ^a^
**156**	H	H	(CH_2_)_11_CH_3_	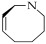	HC=CH	0	n.d. ^a^
**157**	H	H	t-BuOCOMe	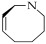	HC=CH	3	n.d. ^a^
**158**	H	H	H	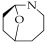	HC=CH	88.4	n.d. ^a^
**159**	OH	H	H	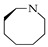	(CH_2_)_2_	29.0	n.d. ^a^
**160**	OH	H	H	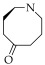	HC=CH	30.0	n.d. ^a^
**161**	H	H	H	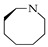	(CH_2_)_2_	0	n.d. ^a^
**162**	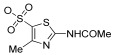	H	H	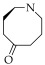	HC=CH	78.8	8.5
**163**	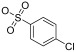	H	H	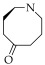	HC=CH	76.7	7.2
**164**	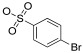	H	H	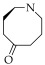	HC=CH	63.0	23.0
**165**	H	H	H	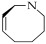	HC=CH	71.2	5.4
**166**	OCOMe	H	H	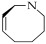	HC=CH	79.1	4.8

^a^ n.d. = not determined.

## Data Availability

Not applicable.
